# Modeling lifetime and count data using a unified flexible family: Its discrete counterpart, properties, and inference

**DOI:** 10.1371/journal.pone.0319091

**Published:** 2025-04-17

**Authors:** Ahmed Z. Afify, Maha M. Helmi, Hassan M. Aljohani, Sara M. A. Alsheikh, Hisham A. Mahran

**Affiliations:** 1 Department of Statistics, Mathematics, and Insurance, Benha University, Benha 13511, Egypt; 2 Department of Mathematics and Statistics, College of Science, Taif University, P.O. Box 11099, Taif 21944, Saudi Arabia; 3 Department of Statistics, Faculty of Science, University of Tabuk, P.O. Box 71491, Tabuk 47512, Saudi Arabia; 4 Department of Statistics, Mathematics and Insurance, Ain Shams University, Cairo 11566, Egypt; University of Malaya, MALAYSIA

## Abstract

In this article, two flexible classes called the modified Kavya–Manoharan-G (MKM-G) and discrete modified Kavya–Manoharan-G (DMKM-G) families are investigated. The two proposed families provide more flexibility for modeling real-lifetime and count data from environmental, medical, engineering, and educational fields. Due to the new extra shape parameter of the two proposed families, their special sub-models are capable of modeling monotonic and non-monotonic hazard rates. The basic properties of the MKM-G family are studied. Eight classical approaches of estimation are used for estimating the MKM-exponential (MKME) parameters. The performances of the estimators are explored using simulation results. Additionally, the DMKM-exponential (DMKME) distribution is defined. Finally, the importance and flexibility of the MKME and DMKME distributions are addressed by fitting seven real-lifetime and count data from aforementioned applied fields. The real data analysis shows that the special models of the two classes are good candidates and can provide close fit as compared to well-known competing continuous and discrete distributions.

## 1 Introduction

Recently, several attempts to develop generalized distributions have been made to model lifetime and count data in different applied fields. These generalized models have several applications including medicine, economics, biological studies, engineering, finance, and environmental sciences, among others. However, there is a clear need for more flexible distributions, which are capable of modeling different shapes of aging and failure criteria.

Some notable families are the Marshall–Olkin-G [1], Kumaraswamy-G [2], Weibull-G [3], Kumaraswamy transmuted-G [4], odd Dagum-G [5], log–logistic tan-G [6], modified generalized-G [7], logarithmic-U [8], Lambert-G [9], new exponential-H [10], and alpha beta-power-F [11], among many others.

One of the recent classes is called the Kavya–Manoharan-G (KM-G) family [12], which is used to introduce some generalized distributions include the KM Kumaraswamy distribution [13], KM log-logistic distribution [14], KM Kumaraswamy exponential distribution [15], KM Burr X distribution [16], KM power Lomax distribution [17].

In this article, we propose a flexible extension of the KM-G class by adding an extra shape parameter. The newly constructed class is called the modified Kavya–Manoharan-G (MKM-G) family, which increases the flexibility of the generated models. Furthermore, the discrete counterpart of the new MKM-G family is proposed. The discrete counterpart class is called the discrete modified Kavya–Manoharan-G (DMKM-G) family. The DMKM-G family is derived using the survival discretization (SD) approach.

The objectives of the current article are five-fold: (i) to propose two new flexible continuous and discrete families; (ii) to present four special lifetime models and a discrete model as special cases of the two proposed classes, with a more detailed analysis of the MKM-exponential (MKME) and DMKM-exponential (DMKME) sub-models; (iii) to address the mathematical characteristics of the MKM-G family; (iv) to discuss the estimation of MKME parameters using eight classical estimation approaches; and (v) to explore the empirical importance of the MKME and DMKME models through analysis of seven real-life datasets, including two count datasets.

We are motivated to introduce the MKM-G and DMKM-G families for several reasons: (i) The MKM-G special models can represent reversed-J shaped, right-skewed, and unimodal densities, as well as bathtub, increasing, modified bathtub, decreasing, and unimodal hazard rate (HR) shapes; (ii) These sub-models generalize several published lifetime models such as the KM Burr X and KM exponential models; (iii) The DMKME distribution exhibits unimodal, reversed-J, increasing, bathtub, and decreasing discrete HR shapes, making it more versatile than other count distributions, which typically only exhibit increasing or decreasing shapes; (iv) The special sub-models of both proposed classes are well-suited for modeling asymmetric lifetime and count data across various applied fields, including insurance, biology, medicine, engineering, and life testing; and (v) The empirical importance of the MKME and DMKME models is explored using seven real-lifetime and count datasets, showing that the two proposed distributions outperform several well-known lifetime and discrete distributions in modeling real-world data; and (vi) Finally, the new families offer simple analytical forms and exceptional flexibility, which may lead to wider applications in engineering, reliability, environmental sciences, insurance, medicine, and economics.

The rest of the paper is outlined in the following eight sections. In Section 2, the MKM-G and DMKM-G families are defined. Five special sub-models of the MKM-G and DMKM-G classes are presented in Section 3. In Section 4, some mathematical properties of the MKM-G class are derived. Estimation methods of the MKME parameters are discussed in Section 5. Detailed simulation results are presented in Section 6. Section 7 provides seven applications for real-lifetime and count data to show the flexibility of the MKME and DMKME distributions. Finally, some concluding remarks are explored in Section 8.

## 2 Synthesis of the MKM-G family and its discrete counterpart

If *X* is a random variable (*RV*) following the KM-G family with parameters *α* and *θ* (see [12]), then it has the following cumulative distribution function (CDF)


$$H\left(x;\vartheta \right)=\psi \left\{1-\text{exp}\left[-G\left(x;\vartheta \right)\right]\right\},x\in ℜ,$$ (1)

where $\psi =\exp\left(1\right)/\left(\text{exp}\left(1\right)-1\right)$ and $G\left(x;\vartheta \right)$ is the baseline CDF with a vector of parameters *ϑ*.

The corresponding probability density function (PDF) of (1) reduces to


$$h\left(x;\vartheta \right)=\psi g\left(x;\varphi \right)\text{exp}\left[-G\left(x;\vartheta \right)\right].$$ (2)

**Definition 1.** A *RV* *X* is said to follow the MKM-G family, denoted by X~MKM-G (θ,ϑ), if its CDF has the form


$$F\left(x;\theta ,\vartheta \right)=\psi \left\{1-\text{exp}\left[-G{\left(x;\vartheta \right)}^{\theta }\right]\right\},x\in ℜ,\theta >0,$$ (3)

where *θ* is a shape parameter.

The corresponding PDF of Equation (3) has the form


$$f\left(x;\theta ,\vartheta \right)=\psi \theta g\left(x;\vartheta \right)G{\left(x;\vartheta \right)}^{\theta -1}\text{exp}\left[-G{\left(x;\vartheta \right)}^{\theta }\right].$$ (4)

The HR function (HRF) of the MKM-G family becomes


$$\varphi \left(x;\theta ,\vartheta \right)=\frac{\psi \theta g\left(x;\vartheta \right)G{\left(x;\vartheta \right)}^{\theta -1}\text{exp}\left[-G{\left(x;\vartheta \right)}^{\theta }\right]}{1-\psi \left\{1-\text{exp}\left[-G{\left(x;\vartheta \right)}^{\theta }\right]\right\}}.$$

The added shape parameter *θ* allows us to explore the tail behavior of the density (4) and provides more flexibility as we can see in Section 3. Additionally, the importance of the MKM-G family follows from its ability to generate new flexible distributions which exhibit monotone and non-monotone failure rates.

**Remark:** The KM-G class follows as a special case by setting θ=1 in Equation (3).

The SD approach is used to discretize the MKM-G family of distributions. The SD technique depends on the survival function (SF), say, $S\left(x\right)=P\left(X\geq x\right),x=1,2,\ldots$ and $S\left(0\right)=1$, which can be adopted to define the probability mass function (PMF) as follows


$$P\left(X=x\right)=S\left(x\right)-S\left(x+1\right),x=0,1,2,\ldots$$ (5)

The SF of the MKM-G family has the form


$$S\left(x;\theta ,\vartheta \right)=1-\psi \left\{1-\exp\left[-G{\left(x;\vartheta \right)}^{\theta }\right]\right\}.$$

Applying the SD approach in (5), the PMF of the DMKM-G family is given by


$$p\left(x;\theta ,\vartheta \right)=\psi \left[q^{G{\left(x;\vartheta \right)}^{\theta }}-q^{G{\left(x+1;\vartheta \right)}^{\theta }}\right],x=0,1,2,\ldots ,\theta >0,$$ (6)

where$q=\text{exp}\left(-1\right).$


$$p\left(x;\theta ,\vartheta \right)=\psi \left\{\text{exp}\left[-G{\left(x;\vartheta \right)}^{\theta }\right]-\text{exp}\left[-G{\left(x+1;\vartheta \right)}^{\theta }\right]\right\}.$$

The CDF and SF of the DMKM-G family is given by


$$F\left(x;\theta ,\vartheta \right)=1-P\left(X\geq x+1\right)=\psi \left[1-q^{G{\left(x+1;\vartheta \right)}^{\theta }}\right]$$

and


$$S\left(x;\theta ,\vartheta \right)=P\left(X\geq x+1\right)=1-\psi \left[1-q^{G{\left(x+1;\vartheta \right)}^{\theta }}\right].$$

## 3 Five special lifetime and discrete models

**Fig 1:**
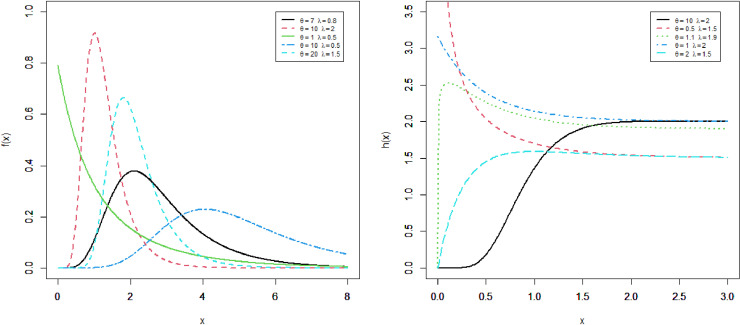
Different shapes of the PDF and HRF of the MKME distribution.

**Fig 2:**
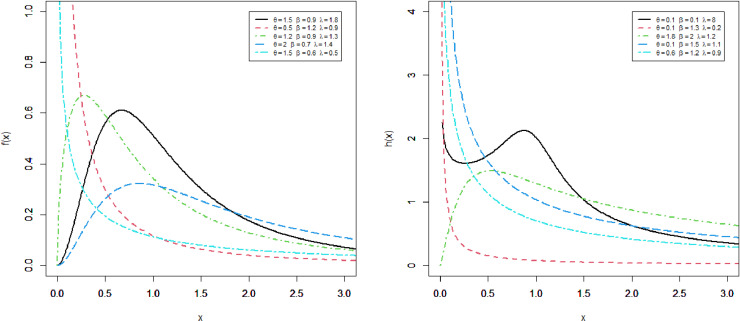
Different shapes of the PDF and HRF of the MKMBXII distribution.

**Fig 3:**
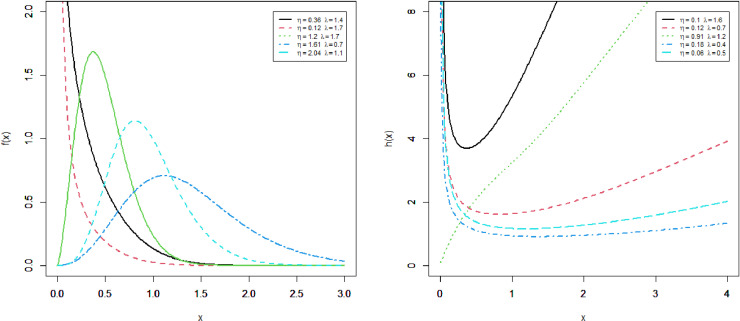
Different shapes of the PDF and HRF of the MKMBx distribution.

**Fig 4:**
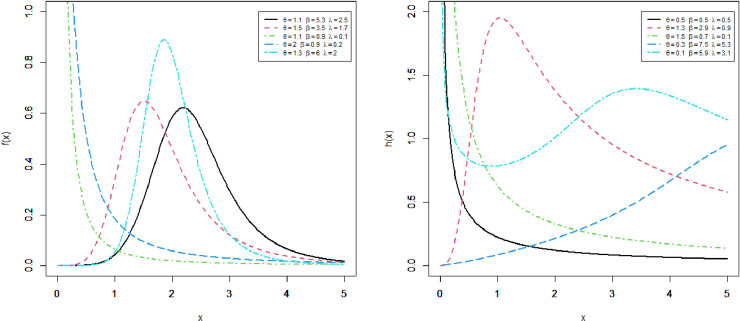
Different shapes of the PDF and HRF of the MKMLL distribution.

**Fig 5:**
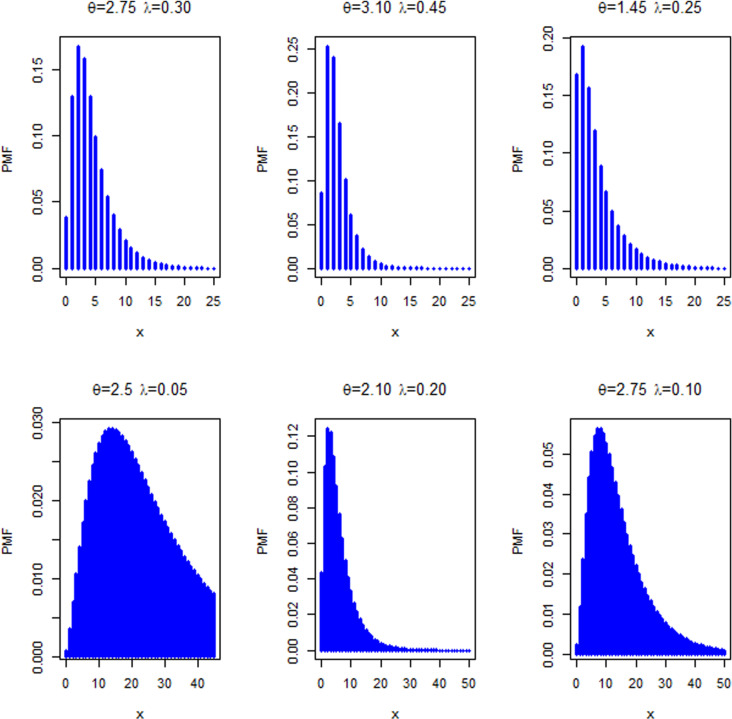
Plots of the PMF of the DMKME distribution for selected values of *θ* and *λ.*

**Fig 6:**
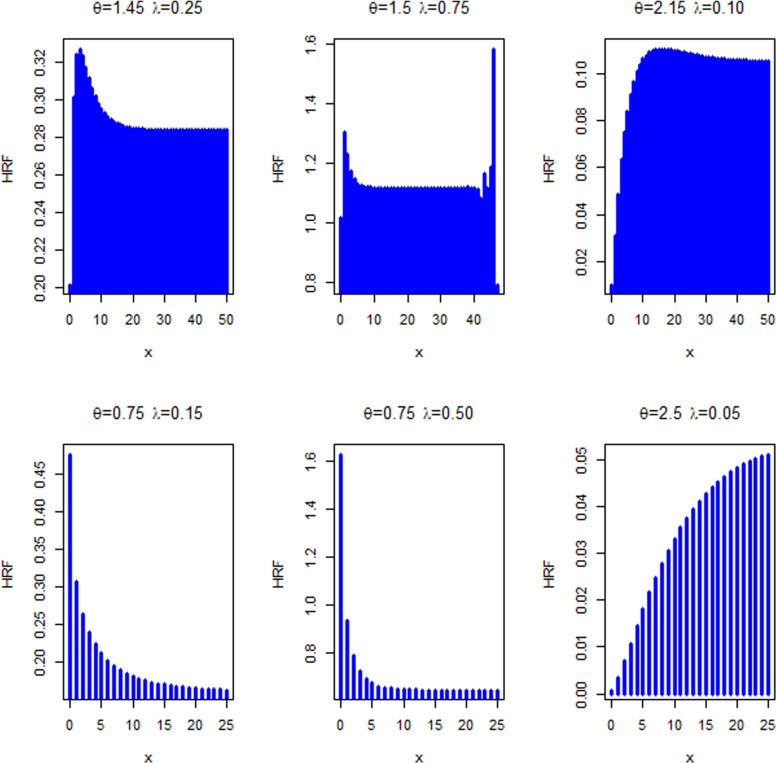
Plots of the HRF of the DMKME distribution for selected values of *θ* and *λ.*

This section presents four special sub-models of the MKM-G family. These sub-models provide flexible forms of some baseline distributions namely the exponential (E), Burr X (Bx), Burr XII (BXII), and log-logistic (LL) distributions. The special sub-models of the MKM-G class are called the MKME, MKM-Burr X (MKMBX), MKM-Burr XII (MKMBXII), and MKM-log logistic (MKMLL) distributions. The four special distributions provide decreasing, bathtub, reversed J shaped, increasing, unimodal, and modified bathtub shapes. Additionally, their densities provide right-skewed, symmetrical, and reversed-J shapes as displayed in Figs 1-4. Furthermore, the DMKME model is defined in Section 3.5. Figs 5 and 6 display the PMF and HRF plots of the DMKME model for different values of its parameters *θ* and *λ*. The HRF of the DMKME distribution provides unimodal, bathtub, increasing, reversed-J, and decreasing discrete failure rates.

### 3.1 The MKME distribution

The CDF of the MKME distribution follows by setting the E CDF, $G\left(x\right)=1-\text{exp}\left(-\lambda x\right),x>0,\lambda >0$, in Equation (3). Then, the CDF of the MKME distribution reduces to


$$F\left(x;\theta ,\lambda \right)=\psi \left(1-\text{exp}\left\{-{\left[1-\text{exp}\left(-\lambda x\right)\right]}^{\theta }\right\}\right),x>0,\theta ,\lambda >0.$$

The corresponding PDF and HRF of the MKME distribution have the forms


$$f\left(x;\theta ,\lambda \right)=\psi \theta \lambda {\left[1-\exp\left(-\lambda x\right)\right]}^{\theta -1}\exp\left\{-\lambda x-{\left[1-\exp\left(-\lambda x\right)\right]}^{\theta }\right\}$$ (7)

and


$$\varphi \left(x;\theta ,\lambda \right)=\frac{\psi \theta \lambda {\left[1-\exp\left(-\lambda x\right)\right]}^{\theta -1}\exp\left\{-\lambda x-{\left[1-\exp\left(-\lambda x\right)\right]}^{\theta }\right\}}{1-\psi \left(1-\text{exp}\left\{-{\left[1-\text{exp}\left(-\lambda x\right)\right]}^{\theta }\right\}\right)}.$$

The *RV* with PDF (7) is denoted by $X\sim \text{MKME}\left(\theta ,\lambda \right)$. For θ=1, the MKME distribution reduces to the KME distribution [12]. Fig 1 gives some possible shapes of the density and HR functions of the MKME distribution.

### 3.2 The MKMBXII Distribution

By taking the CDF of the BXII distribution (for x>0 and β,λ>0), say, $G\left(x\right)=1-{\left(1+x^{\lambda }\right)}^{-\beta }$, as a baseline CDF in (3), the MKMBXII CDF follows as


$$F\left(x;\theta ,\beta ,\lambda \right)=\psi \left(1-\text{exp}\left\{-{\left[1-{\left(1+x^{\lambda }\right)}^{-\beta }\right]}^{\theta }\right\}\right),x>0,\theta ,\beta ,\lambda >0.$$

The PDF of the MKMBXII distribution reduces to


$$f\left(x;\theta ,\beta ,\lambda \right)=\frac{\psi \theta \beta \lambda x^{\lambda -1}}{{\left(1+x^{\lambda }\right)}^{\beta +1}}\text{exp}\left\{-{\left[1-{\left(1+x^{\lambda }\right)}^{-\beta }\right]}^{\theta }\right\}{\left[1-{\left(1+x^{\lambda }\right)}^{-\beta }\right]}^{\theta -1}.$$

For θ=1, the MKMBXII distribution reduces to the KMBXII distribution. Fig 2 gives some shapes of the PDF and HRF of the MKMBXII distribution for different parametric values θ,β and *λ*.

### 3.3 The MKMBx distribution

Consider the CDF of the Bx distribution (for x>0 and λ,β>0), say, $G\left(x\right)={\left\{1-\text{exp}\left[-{\left(\lambda x\right)}^{2}\right]\right\}}^{\beta }$. By inserting the Bx CDF in Equation (3), the CDF of the MKMBx distribution follows as


$$F\left(x;\eta ,\lambda \right)=\psi \left[1-\text{exp}\left(-{\left\{1-\text{exp}\left[-{\left(\lambda x\right)}^{2}\right]\right\}}^{\eta }\right)\right],x>0,\lambda ,\eta >0,$$

where η=θβ.

The PDF of the MKMBx model reduces to


$$f\left(x;\eta ,\lambda \right)=2\psi \eta \lambda^{2}x\text{exp}\left[-{\left(\lambda x\right)}^{2}\right]{\left\{1-\text{exp}\left[-{\left(\lambda x\right)}^{2}\right]\right\}}^{\eta -1}\text{exp}\left(-{\left\{1-\text{exp}\left[-{\left(\lambda x\right)}^{2}\right]\right\}}^{\eta }\right).$$

By setting θ=1 in the above equation, the KMBx distribution [16] follows as a special case. The MKM-Rayleigh distribution is obtained when β=1. The MKMBx model reduces to the KM-Rayleigh distribution for θ=β=1. Some shapes of the density and failure rate functions of the MKMBx distribution are given in Fig 3, for different values of *η* and *λ*.

### 3.4 The MKMLL distribution

Consider the LL CDF (for x>0and β,λ>0), say, $G\left(x\right)=1-{\left[1+{\left(x/\lambda \right)}^{\beta }\right]}^{-1}$. By inserting the LL CDF in Equation (3), we obtain the CDF of the MKMLL distribution (for x>0)


$$F\left(x;\theta ,\beta ,\lambda \right)=\psi \left[1-\text{exp}\left(-{\left\{1-{\left[1+{\left(x/\lambda \right)}^{\beta }\right]}^{-1}\right\}}^{\theta }\right)\right],\theta ,\beta ,\lambda >0.$$

The MKMLL density takes the form


$$f\left(x;\theta ,\beta ,\lambda \right)=\frac{\psi \theta \beta }{\lambda^{\beta }x^{1-\beta }}{\left[1+{\left(\frac{x}{\lambda }\right)}^{\beta }\right]}^{-2}{\left\{1-{\left[1+{\left(x/\lambda \right)}^{\beta }\right]}^{-1}\right\}}^{\theta -1}\text{exp}\left(-{\left\{1-{\left[1+{\left(x/\lambda \right)}^{\beta }\right]}^{-1}\right\}}^{\theta }\right).$$

The MKMLL distribution reduces to the KMLL model for θ=1. Fig 4 provides the shapes of the density and hazard functions of the MKMLL distribution.

### 3.5 The DMKME distribution

The DMKME distribution is derived here by substituting the CDF of the E distribution in Equation (6), then the PMF of the DMKME distribution follows as


$$p\left(x;\theta ,\lambda \right)=\psi \left\{q^{{\left(1-q^{\lambda x}\right)}^{\theta }}-q^{{\left[1-q^{\lambda \left(x+1\right)}\right]}^{\theta }}\right\},x=0,1,2,\ldots ,\theta >0,$$ (8)

where $q=\exp\left(-1\right)$. Then, the corresponding SF and CDF of (8) reduce to


$$S\left(x;\theta ,\lambda \right)=P\left(X\geq x+1\right)=1-\psi \left\{1-q^{{\left[1-q^{\lambda \left(x+1\right)}\right]}^{\theta }}\right\}$$

and


$$F\left(x;\theta ,\lambda \right)=1-P\left(X\geq x+1\right)=\psi \left\{1-q^{{\left[1-q^{\lambda \left(x+1\right)}\right]}^{\theta }}\right\}.$$

The HRF of the DMKME takes the form


$$h\left(x;\theta ,\lambda \right)=\frac{\psi \left\{q^{{\left(1-q^{\lambda x}\right)}^{\theta }}-q^{{\left[1-q^{\lambda \left(x+1\right)}\right]}^{\theta }}\right\}}{1-\psi \left\{1-q^{{\left[1-q^{\lambda \left(x+1\right)}\right]}^{\theta }}\right\}}.$$

The plots of the PMF and HRF of the DMKME model are presented in Figs 5 and 6, for some choices of the parameters *θ* and *λ*. The DMKME HRF can be unimodal, bathtub, increasing and decreasing discrete HRF.

## 4 Properties of the MKM-G family

Some general mathematical properties of the MKM-G family are provided in this section.

### 4.1 Linear representation

Important and simple mixture representations for the CDF and PDF of the MKM-G family in terms of exponentiated-G (Exp-G) density are provided in this section.

Consider the exponential series, which is given by


$$\text{exp}\left(-q\right)=\overset{\infty }{\underset{k=0}{\sum }}{\left(-1\right)}^{k}\frac{q^{k}}{k!}.$$ (9)

Applying (9) to Equation (3), we obtain


$$1-\text{exp}\left[-G{\left(x\right)}^{\theta }\right]=1-\overset{\infty }{\underset{k=0}{\sum }}{\left(-1\right)}^{k}\frac{G{\left(x\right)}^{k\theta }}{k!}.$$

Hence, the MKM-G CDF is expressed as


$$F\left(x\right)=\psi -\overset{\infty }{\underset{k=0}{\sum }}a_{k}H_{k}\left(x\right),$$ (10)

where $a_{k}=\overset{\infty }{\underset{k=0}{\sum }}\psi {\left(-1\right)}^{k}/k!$ And $H_{k}\left(x\right)=G{\left(x\right)}^{k\theta }$ is the Exp-G CDF with power parameter kθ. By differentiating Equation (10), the PDF of the MKM-G family reduces to


$$f\left(x\right)=\overset{\infty }{\underset{k=0}{\sum }}b_{k}h_{k}\left(x\right),$$ (11)

where $b_{k}=-a_{k}$ and $h_{k}\left(x\right)=k\theta g\left(x\right)G{\left(x\right)}^{k\theta -1}$ is the Exp-G PDF with kθ>0. Thus, many mathematical characteristics of the MKM-G family follow simply from those of the Exp-G family.

### 4.2 Quantile function and moments

The quantile function (QF) of the MKM-G family, say, $Q\left(u\right)=F^{-1}\left(x\right)$, follows by inverting (3). Then, the MKM-G QF takes the form


$$Q\left(u\right)=G^{-1}\left({\left\{-\text{log}\left[1-\left(\frac{u}{\psi }\right)\right]\right\}}^{\frac{1}{\theta }}\right),$$

where $G^{-1}\left(\cdot \right)$ ais the baseline QF and $u\in \left(0,1\right)$.

Hereafter, let $Y_{k}$ denotes the Exp-G *RV* with positive power parameter *k*. Hence, based on Equation (11), the *r*th moment of *X* has the form


$$\mu_{r}^{'}=\text{Ε}\left(X^{r}\right)=\overset{\infty }{\underset{k=0}{\sum }}b_{k}E\left(Y_{k}^{r}\right).$$ (12)

Setting r=1 in Equation (12) gives the mean of *X* ($\mu_{1}^{'}$).

The moment generating function (MGF) of the *RV* *X* is defined by $M_{X}\left(t\right)=E\left(e^{tX}\right)$. Hence, the MGF of the MKM-G family can be derived from (11) in two different forms. The first one follows as


$$M_{X}\left(t\right)=\overset{\infty }{\underset{k=0}{\sum }}b_{k}M_{k}\left(t\right),$$

where $M_{k}\left(t\right)$ is the MGF of $Y_{k}\left(x\right)$. Then, $M_{X}\left(t\right)$ of the MKM-G family is calculated based on the MGF of the Exp-G class.

The second formula for $M_{X}\left(t\right)$ takes the form


$$M_{X}\left(t\right)=\overset{\infty }{\underset{k=0}{\sum }}kb_{k}\varphi \left(t,k-1\right),$$

where $\varphi \left(t,k-1\right)=\overset{1}{\underset{0}{\int }}u^{k-1}\text{exp}\left[tQ_{G}\left(u\right)\right]du$.

The *q*th incomplete moment of *X* is expressed based on (11) as


$$\delta_{q}\left(t\right)=\overset{t}{\underset{-\infty }{\int }}x^{q}f\left(x\right)dx=\overset{\infty }{\underset{k=0}{\sum }}b_{k}\overset{t}{\underset{-\infty }{\int }}x^{q}h_{k}\left(x\right)dx.$$ (13)

The first incomplete moment (FIM), say, $\delta_{1}\left(t\right)$, follows from (13) when q=1. It also can be calculated by two formulae. The first formula for $\delta_{1}\left(t\right)$ follows from (13) as


$$\delta_{1}\left(t\right)=\overset{\infty }{\underset{k=0}{\sum }}b_{k}\gamma_{k}\left(t\right),$$ (14)

where $\gamma_{k}\left(t\right)=\overset{t}{\underset{-\infty }{\int }}xh_{k}\left(x\right)dx$ is the FIM of the Exp-G class. The second formula for $\delta_{1}\left(t\right)$ reduces to


$$\delta_{1}\left(t\right)=\overset{\infty }{\underset{k=0}{\sum }}b_{k}\rho_{k}\left(t\right),$$

where $\rho_{k}\left(t\right)=\overset{G\left(t\right)}{\underset{0}{\int }}ku^{k-1}G^{-1}\left(u\right)du$, where $\rho_{k}\left(t\right)$ is computed numerically and $G^{-1}\left(u\right)$ is the baseline QF.

Bonferroni ($B\left(\tau \right)$) and Lorenz $\left(L\left(\tau \right)\right)$ curves are useful applications for $\delta_{1}\left(t\right)$ because they can be calculated, for a given probability *τ*, by $B\left(\tau \right)=\delta_{1}\left(d\right)/\left(\tau \mu_{1}^{'}\right)$ and $L\left(\tau \right)=\delta_{1}\left(d\right)/\mu_{1}^{'}$, where $\mu_{1}^{'}$ is the mean of *X* and $d=Q\left(\tau \right)$ is the QF of *X* at *τ*. The two curves have important applications in demography, economics, insurance, reliability, and medicine. Additionally, the mean deviations about the mean $\left[\varphi_{1}=2\mu_{1}^{'}F\left(\mu_{1}^{'}\right)-2\delta_{1}\left(\mu_{1}^{'}\right)\right]$ and about the median $\left[\varphi_{2}=\mu_{1}^{'}-2\delta_{1}\left(M\right)\right]$ of *X*, where$M=Q\left(0.5\right)$ is the median and $F\left(\mu_{1}^{'}\right)$ is simply evaluated from (3).

### 4.3 Entropies

The variation of the uncertainty can be measured by the Rényi entropy, say $I_{\phi }$, which is given by


$$I_{\phi }=\frac{1}{1-\phi }\log\left(\overset{\infty }{\underset{-\infty }{\int }}f{\left(x\right)}^{\phi }dx\right),\phi >0\text{ and }\phi \neq 1.$$

Using the MKM-G density (4), we obtain


$$f{\left(x\right)}^{\phi }={\left(\theta \psi \right)}^{\phi }g{\left(x\right)}^{\phi }\exp\left[-G{\left(x\right)}^{\phi \theta }\right]G{\left(x\right)}^{\phi \left(\theta -1\right)}.$$

By applying the power series in Equation (9), we have


$$\exp\left[-G{\left(x\right)}^{\phi \theta }\right]=\overset{\infty }{\underset{k=0}{\sum }}\frac{{\left(-1\right)}^{k}}{k!}G{\left(x\right)}^{\phi k\theta }.$$

Then, $f{\left(x\right)}^{\phi }$ reduces to


$$f{\left(x\right)}^{\phi }={\left(\theta \psi \right)}^{\phi }\overset{\infty }{\underset{k=0}{\sum }}\frac{{\left(-1\right)}^{k}}{k!}g{\left(x\right)}^{\phi }G{\left(x\right)}^{\phi \left(k\theta +\theta -1\right)}.$$

Hence, $f{\left(x\right)}^{\phi }$ follows as


$$f{\left(x\right)}^{\phi }=\overset{\infty }{\underset{k=0}{\sum }}s_{k}g{\left(x\right)}^{\phi }G{\left(x\right)}^{\phi \left(k\theta +\theta -1\right)},$$

where$s_{k}={\left(\theta \psi \right)}^{\phi }{\left(-1\right)}^{k}/k!.$

Then, the Rényi entropy of the MKM-G family reduces to


$$I_{\phi }=\frac{1}{1-\phi }\log\left[\overset{\infty }{\underset{k=0}{\sum }}s_{k}\overset{\infty }{\underset{-\infty }{\int }}g{\left(x\right)}^{\phi }G{\left(x\right)}^{\phi \left(k\theta +\theta -1\right)}dx\right].$$

The Shannon entropy follows from the Rényi entropy when *ϕ* tends to 1.

### 4.4 Mean residual life and mean inactivity time

The expected additional life length for a unit, which is alive at age *t* can be expressed by the mean residual life (MRL). The MRL of a *RV* *X* is defined (for t>0) by $M_{X}\left(t\right)=E\left(X-t\text{|}X>t\right)$. The MRL of *X* reduces to


$$M_{X}\left(t\right)=\frac{\left[1-\delta_{1}\left(t\right)\right]}{1-F\left(t\right)}-t,$$ (15)

where $F\left(t\right)$ is the CDF of the MKM-G family. Inserting (14) in Equation (15) gives


$$M_{X}\left(t\right)=\frac{1}{1-F\left(t\right)}\left[1-\overset{\infty }{\underset{k=0}{\sum }}b_{k}\gamma_{k}\left(t\right),\right]-t.$$

The waiting time elapsed since the failure of an item on condition that this failure had occurred in $\left(0,t\right)$ is expressed as the mean inactivity time (MIT). The MIT of *X*, say ${\text{Φ}}_{X}\left(t\right)=E\left(t-X\text{|}X\leq t\right),$ follows as


$${\text{Φ}}_{X}\left(t\right)=t-\frac{\delta_{1}\left(t\right)}{F\left(t\right)}.$$

Combining the last equation and Equation (14), the MIT of *X* takes the form


$${\text{Φ}}_{X}\left(t\right)=t-\frac{1}{F\left(t\right)}\overset{\infty }{\underset{k=0}{\sum }}b_{k}\gamma_{k}\left(t\right).$$

### 4.5 Probability weighted moments

The expectation of a certain function of a *RV* whose mean exists is known as the probability weighted moments (PWMs). The $\left(j,i\right)$th PWM of *X* has the form


$$\rho_{j,i}=E\left\{X^{j}F{\left(X\right)}^{i}\right\}=\overset{\infty }{\underset{-\infty }{\int }}x^{j}F{\left(x\right)}^{i}f\left(x\right)dx.$$

Using the CDF and PDF of the MKM-G family, we can write


$$F{\left(x\right)}^{i}f\left(x\right)=\theta \psi^{i-1}g\left(x\right)\exp\left[-G{\left(x\right)}^{\theta }\right]G{\left(x\right)}^{\theta -1}{\left\{1-\exp\left[-G{\left(x\right)}^{\theta }\right]\right\}}^{i}.$$

Applying the exponential and binomial series, the above equation reduces to


$$F{\left(x\right)}^{i}f\left(x\right)=\theta \psi^{i+1}\overset{\infty }{\underset{k,s,l=0}{\sum }}\frac{{\left(-1\right)}^{k+s+l}}{s!l!}\left(\begin{array}{c} i\\ k \end{array}\right)g\left(x\right)G{\left(x\right)}^{\theta \left(lk+s+1\right)-1}.$$ (16)

Equivalently, we can write


$$F{\left(x\right)}^{i}f\left(x\right)=\overset{\infty }{\underset{k,s,l=0}{\sum }}m_{k,s,l}h_{\theta \left(lk+s+1\right)}\left(x\right),$$

where


$$m_{k,s,l}=\frac{\theta \psi^{i+1}{\left(-1\right)}^{k+s+l}}{s!l!\left[\theta \left(lk+s\right)+\theta \right]}\left(\begin{array}{c} i\\ k \end{array}\right).$$

Then, the PWM of the MKM-G family follows as


$$\rho_{j,i}=\overset{\infty }{\underset{k,s,l=0}{\sum }}m_{k,s,l}\overset{\infty }{\underset{-\infty }{\int }}x^{j}h_{\theta \left(lk+s+1\right)}\left(x\right)dx=\overset{\infty }{\underset{k,s,l=0}{\sum }}m_{k,s,l}E\left(Y_{\theta \left(lk+s+1\right)}^{j}\right).$$

### 4.6 Order statistics

Let $X_{1},\ldots ,X_{n}$ be a random sample from the MKM-G family. The density of the *i*th order statistic, say $X_{i:n}$, has the form


$$f_{i:n}\left(x\right)=\frac{f\left(x\right)}{B\left(i,n-i+1\right)}\overset{n-i}{\underset{j=0}{\sum }}{\left(-1\right)}^{j}\left(\begin{array}{c} n-1\\ j \end{array}\right)F{\left(x\right)}^{j+i-1},$$ (17)

where $B\left(.,.\right)$ is the beta function.

Using Equations (3) and (4) of the MKM-G family, we obtain


$$F{\left(x\right)}^{j+i-1}f\left(x\right)=\psi^{j+i}\theta g\left(x\right)\text{exp}\left[-G{\left(x\right)}^{\theta }\right]G{\left(x\right)}^{\theta -1}{\left\{1-\text{exp}\left[-G{\left(x\right)}^{\theta }\right]\right\}}^{j+i-1}.$$

Based on Equation (16), the last equation has the form


$$F{\left(x\right)}^{j+i-1}f\left(x\right)=\overset{\infty }{\underset{k,s,l=0}{\sum }}C_{k,s,l}h_{\theta \left(lk+s+1\right)}\left(x\right),$$ (18)

where $h_{\theta \left(lk+s+1\right)}\left(x\right)$ is the Exp-G PDF with parameter $\theta \left(lk+s\right)+\theta$ and


$$C_{k,s,l}=\left(\begin{array}{c} j+i-1\\ k \end{array}\right)\frac{\theta \psi^{j+i}{\left(-1\right)}^{k+s+l}}{s!l!\left[\theta \left(lk+s\right)+\theta \right]}.$$

Then, the PDF of $X_{i:n}$ is expressed by


$$f_{i:n}\left(x\right)=\overset{\infty }{\underset{k,s,l=0}{\sum }}\overset{n-i}{\underset{j=0}{\sum }}\left(\begin{array}{c} n-1\\ j \end{array}\right)\frac{{\left(-1\right)}^{j}C_{k,s,l}}{B\left(i,n-i+1\right)}h_{\theta \left(lk+s+1\right)}\left(x\right).$$ (19)

Hence, the PDF of the MKM-G order statistic is a linear combination of Exp-G densities. Equation (19) illustrates that the properties of $X_{i:n}$ can be derived from those properties of $Y_{\theta \left(lk+s+1\right)}$.

The *r*th moment of $X_{i:n}$ follows as


$$E\left(X_{i:n}^{r}\right)=\overset{\infty }{\underset{k,s,l=0}{\sum }}\overset{n-i}{\underset{j=0}{\sum }}\frac{{\left(-1\right)}^{j}C_{k}}{B\left(i,n-i+1\right)}\left(\begin{array}{c} n-1\\ j \end{array}\right)E\left(Y_{\theta \left(lk+s+1\right)}^{r}\right).$$

### 4.7 Moments of the MKME distribution

This subsection provides a simple expression for the *r*th moment of the MKME model.

Based on Equation (11), the PDF of the MKME distribution reduces to


$$f\left(x\right)=\overset{\infty }{\underset{k=0}{\sum }}b_{k}k\theta \lambda {\left[1-\text{exp}\left(-\lambda x\right)\right]}^{k\theta -1}\text{exp}\left(-\lambda x\right).$$

Applying the binomial expansion to the last term, the above equation becomes


$$f\left(x\right)=\overset{\infty }{\underset{m=0}{\sum }}v_{m}g_{m+1}\left(x\right),$$ (20)

where


$$v_{m}=\overset{\infty }{\underset{k=0}{\sum }}\frac{\psi k\theta {\left(-1\right)}^{k+m+1}}{\left(m+1\right)\left(k\right)!}\left(\begin{array}{c} k\theta -1\\ m \end{array}\right)$$

and $g_{m+1}\left(x\right)$ is the PDF of the E model with scale parameter $\left(m+1\right)\lambda$. Then, the MKME PDF is expressed as a single linear combination of E PDFs.

The *r*th moment of the MKME distribution is obtained from Equation (20) as follows


$$\mu_{r}^{'}=r!\overset{\infty }{\underset{m=0}{\sum }}v_{m}{\left[\left(m+1\right)\lambda \right]}^{-r}.$$ (21)

The mean of X, say, $\mu_{x}$, follows from (21) with r=1. Furthermore, the R software is used to obtain some numerical values for the $\mu_{x}$, variance $\left(\sigma_{x}^{2}\right)$, skewness $\left(SK\right)$, and kurtosis $\left(KU\right)$ measures of the MKME distribution. The values of the given measures are reported in Table 1 It is noted that that the spread for its KU is much larger ranging from 6.6978 to 18.6635, whereas the SK of the MKME distribution can range in the interval (1.4482, 3.2662).

**Table 1:** The values of some measures of the MKME distribution for several values of *θ* and *λ.*

*λ*	*θ*	$\mu_{x}$	$\sigma_{x}^{2}$	SK	KU
0.2	0.5	2.1863	12.4411	3.2662	18.6635
	0.75	3.0696	16.0281	2.7305	14.1281
	1.5	5.1025	22.2029	2.0990	9.8540
	5	9.8791	29.7206	1.5725	7.1999
	10	13.0473	31.9048	1.4482	6.6978
0.5	0.5	0.8745	1.9906	3.2662	18.6635
	0.75	1.2278	2.5645	2.7305	14.1281
	1.5	2.0410	3.5525	2.0990	9.8540
	5	3.9517	4.7553	1.5725	7.1999
	10	5.2189	5.1048	1.4482	6.6978
0.75	0.5	0.5830	0.8847	3.2662	18.6635
	0.75	0.8186	1.1398	2.7305	14.1281
	1.5	1.3607	1.5789	2.0990	9.8540
	5	2.6344	2.1135	1.5725	7.1999
	10	3.4793	2.2688	1.4482	6.6978
1.5	0.5	0.2915	0.2212	3.2662	18.6635
	0.75	0.4093	0.2849	2.7305	14.1281
	1.5	0.6803	0.3947	2.0990	9.8540
	5	1.3172	0.5284	1.5725	7.1999
	10	1.7396	0.5672	1.4482	6.6978
2	0.5	0.2186	0.1244	3.2662	18.6635
	0.75	0.3070	0.1603	2.7305	14.1281
	1.5	0.5102	0.2220	2.0990	9.8540
	5	0.9879	0.2972	1.5725	7.1999
	10	1.3047	0.3190	1.4482	6.6978

Additionally, Table 2 provides the numerical values of $\mu_{X}$ for the MKME model based on the numerical integration (NUI) and summation (SUM) formula for several values of *λ* and *θ* at truncated *M* terms, where *L* is the truncated terms from this summation. Table 2 shows that the summation in (21) converges to the NUI of $\mu_{X}$ for all values of *λ* and *θ* when *L* reaches to 50.

**Table 2:** The mean of the MKME model based on the SUM and NUI formulae for some values of *λ* and *θ* at truncated *M* terms.

*λ*	*θ*	*M*	SUM	NUI
0.5	0.5	10	0.8592	0.8745
		15	0.8656	
		25	0.8702	
		50	0.8729	
	0.8	10	1.2880	1.2925
		15	1.2903	
		25	1.2916	
		50	1.2922	
	2.5	10	2.7412	2.7898
		15	2.7893	
		25	2.7898	
		50	2.7898	
0.9	0.5	10	0.4773	0.4859
		15	0.4809	
		25	0.4834	
		50	0.4850	
	0.8	10	0.7156	0.7181
		15	0.7168	
		25	0.7176	
		50	0.7179	
	2.5	10	1.5229	1.5499
		15	1.5496	
		25	1.5499	
		50	1.5499	
1.5	0.5	10	0.2864	0.2915
		15	0.2885	
		25	0.2901	
		50	0.2910	
	0.8	10	0.4293	0.4308
		15	0.4301	
		25	0.4305	
		50	0.4308	
	2.5	10	0.9137	0.9299
		15	0.9298	
		25	0.9299	
		50	0.9299	

**Fig 7:**
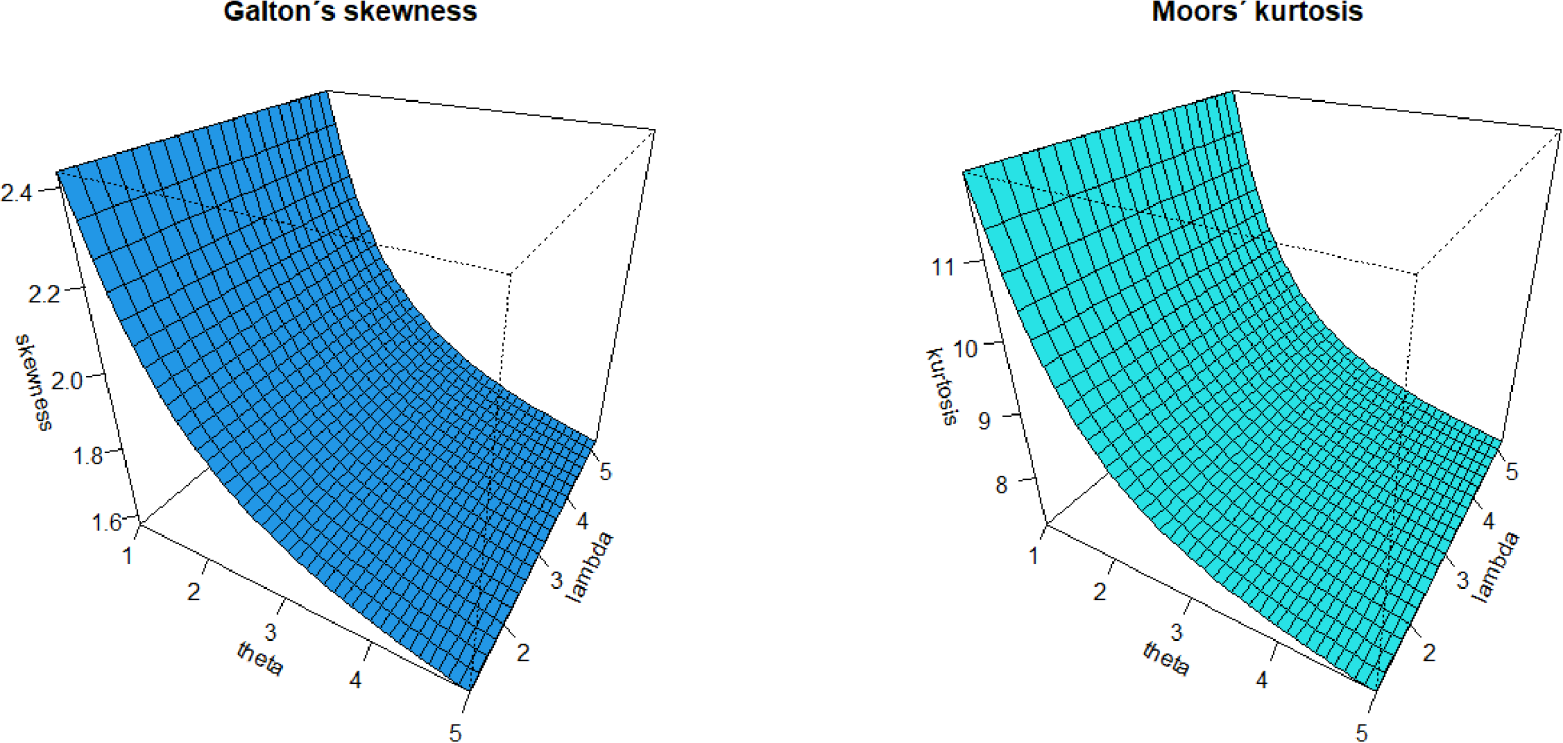
Plots of the GS and MK for the MKME distribution.

The Galton´s skewness (GS) and Moors´ kurtosis (MK) are calculated based on the QF. Fig. 7 displays the GS and MK of the MKME distribution for some parametric values of *λ* and *θ*.

The QF of the MKME distribution is


$$Q\left(u\right)=\frac{-1}{\lambda }\text{log}\left\{1-{\left(-\text{log}\left(1-\frac{u}{\psi }\right)\right)}^{\frac{1}{\theta }}\right\}.$$

## 5 Estimation approaches

This section discusses the estimation of the MKME parameters using some classical methods called the maximum likelihood (ML), least-squares (LS), weighted least-squares (WLS), maximum product of spacing (MPS), percentiles (PC), Cramér–von Mises (CM), Anderson–Darling (AD), and right-tail AD (RTAD) estimators.

Let $x_{1},\ldots ,x_{n}$ be a random sample from the MKME distribution and $x_{1:n}<x_{2:n}<\ldots <x_{n:n}$ be their order statistics. The log-likelihood function, say, *l*, reduces to


$$l=n\log\left(\psi \right)+n\log\theta +n\log\lambda -\overset{n}{\underset{i=1}{\sum }}p_{i}^{\theta }-\lambda \overset{n}{\underset{i=1}{\sum }}x_{i}+\left(\theta -1\right)\overset{n}{\underset{i=1}{\sum }}\log\left(p_{i}\right),$$

where$p_{i}=1-\exp\left(-\lambda x_{i}\right).$

The ML estimators (MLEs) of *θ* and *λ* are determined by maximizing the above equation with respect to the parameters *θ* and *λ*, or by solving the following two equations


$$\frac{\partial l}{\partial \theta }=\frac{n}{\theta }-\overset{n}{\underset{i=1}{\sum }}p_{i}^{\theta }\text{log}\left(p_{i}\right)+\overset{n}{\underset{i=1}{\sum }}\log\left(p_{i}\right)=0$$

and


$$\frac{\partial l}{\partial \lambda }=\frac{n}{\lambda }-\overset{n}{\underset{i=1}{\sum }}x_{i}-\theta \overset{n}{\underset{i=1}{\sum }}x_{i}\left(1-p_{i}\right)p_{i}^{\theta -1}+\left(\theta -1\right)\overset{n}{\underset{i=1}{\sum }}\frac{x_{i}\left(1-p_{i}\right)}{p_{i}}=0.$$

The MLEs are also calculated by using different statistical programs such as Mathematica, Mathcad, and R (optim function), among others.

Swain et al. [18] estimated the parameters of the beta distribution using the LS estimators (LSEs) and WLS estimators (WLSEs). The LSEs and WLSEs of the MKME parameters *θ* and *λ* are determined by minimizing


$$V\left(\theta ,\lambda \right)=\overset{n}{\underset{i=1}{\sum }}d_{i}{\left\{\psi -\psi \exp\left(-p_{i:n}^{\theta }\right)-\frac{i}{n+1}\right\}}^{2},$$

where $d_{i}=1$ for the LS approach, $d_{i}=\frac{{\left(n+1\right)}^{2}\left(n+2\right)}{\left[i\left(n-i+1\right)\right]}$ for the WLS approach and$p_{i:n}=1-\exp\left(-\lambda x_{i:n}\right).$

Furthermore, the LSEs and WLSEs can be obtained by solving the following nonlinear equations


$$\overset{n}{\underset{i=1}{\sum }}d_{i}\left[\psi -\psi \exp\left(-p_{i:n}^{\theta }\right)-\frac{i}{n+1}\right]\Delta_{l}\left(x_{i:n}\text{|}\theta ,\lambda \right)=0,l=1,2,$$

where


$$\Delta_{1}\left(x_{i:n}\text{|}\theta ,\lambda \right)=\frac{\partial l}{\partial \theta }F\left(x_{i:n}\text{|}\theta ,\lambda \right)=\psi p_{i:n}^{\theta }\exp\left(-p_{i:n}^{\theta }\right)\text{log}\left(p_{i:n}\right)$$ (22)

and


$$\Delta_{2}\left(x_{i:n}\text{|}\theta ,\lambda \right)=\frac{\partial l}{\partial \lambda }F\left(x_{i:n}\text{|}\theta ,\lambda \right)=\theta x_{i:n}\exp\left(-\lambda x_{i:n}\right)p_{i:n}^{\theta -1}\psi \exp\left(-p_{i:n}^{\theta }\right).$$ (23)

Cheng and Amin [19, 20] pioneered the MPS approach as an alternative method to estimate the parameters of different continuous univariate models. The uniform spacings, say $D_{i}$, of a random sample of size *n* from the MKME distribution are defined by


$$D_{i}\left(\theta ,\lambda \right)=F\left(x_{i:n}\text{|}\theta ,\lambda \right)-F\left(x_{i-1:n}\text{|}\theta ,\lambda \right),$$

where $F\left(x_{0:n}\text{|}\theta ,\lambda \right)=0,\overset{n+1}{\underset{i=1}{\sum }}D_{i}\left(\theta ,\lambda \right)=1$ and $F\left(x_{n+1:n}\text{|}\theta ,\lambda \right)=1.$

The MPS estimators (MPSEs) of the MKME parameters can be determined by maximizing


$$G\left(\theta ,\lambda \right)=\frac{1}{n+1}\overset{n+1}{\underset{i=1}{\sum }}\log D_{i}\left(\theta ,\lambda \right),$$

with respect to *θ* and *λ*.

Additionally, the MPSEs of the MKME parameters are also calculated by solving


$$\frac{1}{n+1}\overset{n+1}{\underset{i=1}{\sum }}\frac{1}{D_{i}\left(\theta ,\lambda \right)}\left[\Delta_{s}\left(x_{i:n}\text{|}\theta ,\lambda \right)-\Delta_{l}\left(x_{i-1:n}\text{|}\theta ,\lambda \right)\right]=0,l=1,2,$$

where $l\left(x_{i:n}\text{|}\theta ,\lambda \right)=0$are defined in Equations (22) and (23) for l=1,2.

The PC estimators (PCEs) are proposed by [21] to estimate the model parameters by equating the sample PC points with the population PC points. If $p_{i}=i/\left(n+1\right)$ is an unbiased estimator of $F\left(x_{i:n}\text{|}\theta ,\lambda \right)$, then the PCEs of the parameters of the MKME distribution follow by minimizing


$$P\left(\theta ,\lambda \right)=\overset{n}{\underset{i=1}{\sum }}{\left(x_{i:n}+\frac{1}{\lambda }\text{log}\left\{1-{\left[-\log\left(1-\frac{p_{i}}{\psi }\right)\right]}^{\frac{1}{\theta }}\right\}\right)}^{2},$$

with respect to *θ* and *λ*.

Cramér [22] and Von Mises [23] introduced the CVM estimators (CVMEs) which are obtained as the difference between the estimated CDF and empirical CDF.

The CVMEs of the MKME parameters can be determined by minimizing


$$C\left(\theta ,\lambda \right)=\frac{1}{12n}+\overset{n}{\underset{i=1}{\sum }}{\left[\psi -\psi \exp\left(-p_{i:n}^{\theta }\right)-\frac{2i-1}{2n}\right]}^{2}.$$

Furthermore, the CVMEs are also obtained by solving the following nonlinear equations


$$\overset{n}{\underset{i=1}{\sum }}\left[\psi -\psi \exp\left(-p_{i:n}^{\theta }\right)-\frac{2i-1}{2n}\right]\Delta_{l}\left(x_{i:n}\text{|}\theta ,\lambda \right)=0,\text{ for }l=1,2$$

The AD estimators (ADEs) are an important type of minimum distance estimators.

The ADEs of the MKME parameters can be determined by minimizing


$$A\overset{n}{\underset{i=1}{\sum }}\left[\psi -\psi \exp\left(-p_{i:n}^{\theta }\right)-\frac{2i-1}{2n}\right]\Delta_{l}\left(x_{i:n}\text{|}\theta ,\lambda \right)=0,$$

with respect to *θ* and *λ*. The ADEs are also calculated by solving the following equations


$$\overset{n}{\underset{i=1}{\sum }}\left(2i-1\right)\left[\frac{\Delta_{s}\left(x_{i:n}\text{|}\theta ,\lambda \right)}{F\left(x_{i:n}\text{|}\theta ,\lambda \right)}-\frac{\Delta_{j}\left(x_{n+1-i:n}\text{|}\theta ,\lambda \right)}{S\left(x_{n+1-i:n}\text{|}\theta ,\lambda \right)}\right]=0.$$

The RTAD estimators (RTADEs) of *θ* and *λ* are given by minimizing


$$R\left(\theta ,\lambda \right)=\frac{n}{2}-2\overset{n}{\underset{i=1}{\sum }}F\left(x_{i:n}\text{|}\theta ,\lambda \right)-\frac{1}{n}\overset{n}{\underset{i=1}{\sum }}\left(2i-1\right)\log\bar{F}\left(x_{n+1-i:n}\text{|}\theta ,\lambda \right).$$

## 6 Simulation results

This section explores the performance of several estimators of the MKME parameters by using detailed simulation studies. We generate 5000 samples from the MKME distribution based on some sample sizes $n=\left\{20,50,100,250\right\}$ and some parametric values of the two parameters $\theta =\left(0.5,0.75,1.5,2\right)$ and $\lambda =\left(0.5,1.3,1.5\right)$. We calculate the average estimates (AEs) of the parameters and mean square errors (MSEs) for each sample size using the eight estimators.

The performance of the studied estimators is evaluated using MSEs. The AEs and MSEs (in parentheses) for different estimators are presented in Tables 3–6. It is observed that as the sample size *n* increases, the AEs converge to the true parameter values, and the MSEs decrease towards zero, demonstrating that the estimators are asymptotically unbiased. Overall, the numerical simulations show that all eight estimation methods perform excellently with respect to the MSEs.

**Table 3:** The AEs and MSEs of the MKME parameters for n=20.

*θ*	*λ*	MLEs	LSEs	WLSEs	MPSEs	PCEs	CVMEs	ADEs	RTADEs
0.25	0.50	0.26580(0.0016)	0.23958(0.0019)	0.24304(0.0016)	0.22771(0.0016)	0.22555(0.0137)	0.26144(0.0019)	0.24888(0.0014)	0.25578(0.0018)
		0.58768(0.0369)	0.42586(0.0655)	0.45038(0.0509)	0.36540(0.0481)	0.38996(0.0818)	0.58373(0.0673)	0.48136(0.0436)	0.50868(0.0428)
	0.85	0.26597(0.0015)	0.24299(0.0018)	0.24624(0.0015)	0.23077(0.0014)	0.23584(0.0126)	0.26058(0.0018)	0.25000(0.0015)	0.25188(0.0018)
		1.02263(0.1057)	0.72155(0.1662)	0.76418(0.1590)	0.62825(0.1330)	0.67845(0.2137)	0.93337(0.1825)	0.84035(0.1202)	0.84189(0.1188)
	1.30	0.26179(0.0013)	0.24276(0.0017)	0.24239(0.0016)	0.23013(0.0015)	0.23194(0.0125)	0.25774(0.0019)	0.25113(0.0014)	0.25385(0.0017)
		1.54300(0.2680)	1.14200(0.4040)	1.12700(0.3650)	0.96900(0.3140)	1.01200(0.5100)	1.48700(0.4360)	1.27600(0.2900)	1.29100(0.2690)
	1.50	0.26214(0.0013)	0.24125(0.0018)	0.24707(0.0015)	0.22648(0.0016)	0.23024(0.0135)	0.26096(0.0020)	0.25045(0.0013)	0.25413(0.0018)
		1.75299(0.3123)	1.18251(0.5388)	1.33528(0.4493)	1.08439(0.4454)	1.14602(0.6299)	1.67127(0.5354)	1.50946(0.3603)	1.55412(0.3433)
	2.50	0.25752(0.0012)	0.24178(0.0018)	0.24547(0.0014)	0.23035(0.0015)	0.23078(0.0119)	0.26186(0.0018)	0.24828(0.0014)	0.25120(0.0018)
		2.81960(0.8080)	2.08206(1.5536)	2.23315(1.1861)	1.82685(1.1468)	1.96722(1.3057)	2.73010(1.4913)	2.39454(1.0055)	2.51822(0.8592)
0.50	0.50	0.52770(0.0067)	0.47949(0.0086)	0.48844(0.0077)	0.45087(0.0073)	0.44602(0.0322)	0.53294(0.0101)	0.49359(0.0074)	0.51369(0.0088)
		0.55232(0.0212)	0.45157(0.0353)	0.46447(0.0271)	0.40139(0.0276)	0.40815(0.0464)	0.54992(0.0325)	0.48916(0.0246)	0.50825(0.0239)
	0.85	0.53132(0.0063)	0.47595(0.0085)	0.48352(0.0076)	0.44735(0.0080)	0.46521(0.0335)	0.52119(0.0086)	0.50573(0.0069)	0.50878(0.0086)
		0.96097(0.0615)	0.76390(0.0890)	0.76917(0.0769)	0.67396(0.0773)	0.71865(0.1278)	0.90734(0.0984)	0.85817(0.0667)	0.86685(0.0704)
	1.30	0.52958(0.0065)	0.47764(0.0083)	0.48658(0.0079)	0.44749(0.0076)	0.46233(0.0328)	0.53317(0.0095)	0.49974(0.0059)	0.51130(0.0093)
		1.43800(0.1490)	1.18700(0.2190)	1.19900(0.2040)	1.00700(0.1920)	1.02400(0.2880)	1.42100(0.2200)	1.27300(0.1620)	1.3000(0.1600)
	1.50	0.53195(0.0062)	0.48087(0.0091)	0.49123(0.0081)	0.44820(0.0081)	0.44922(0.0306)	0.52068(0.0084)	0.49528(0.0066)	0.50637(0.0091)
		1.63679(0.1911)	1.35738(0.2721)	1.39823(0.2391)	1.17979(0.2506)	1.20761(0.3764)	1.63628(0.2906)	1.45935(0.2132)	1.54610(0.2329)
	2.50	0.52636(0.0065)	0.48124(0.0083)	0.49023(0.0091)	0.45141(0.0084)	0.46360(0.0312)	0.51189(0.0097)	0.49507(0.0066)	0.51182(0.0093)
		2.84736(0.6121)	2.24796(0.7508)	2.36092(0.6947)	1.96700(0.7025)	2.01568(1.1273)	2.63756(0.8255)	2.45082(0.6000)	2.56827(0.5527)
0.75	0.5	0.79313(0.0169)	0.71322(0.0241)	0.71616(0.0204)	0.66444(0.0182)	0.69061(0.0545)	0.79517(0.0231)	0.74407(0.0192)	0.77383(0.0247)
		0.54462(0.0169)	0.46171(0.0249)	0.47279(0.0201)	0.40664(0.0196)	0.42749(0.0288)	0.53473(0.0221)	0.48520(0.0163)	0.50815(0.0184)
	0.85	0.80609(0.0177)	0.71729(0.0225)	0.72418(0.0209)	0.67217(0.0200)	0.69346(0.0583)	0.79700(0.0225)	0.75488(0.0186)	0.76784(0.0242)
		0.92533(0.0497)	0.78162(0.0650)	0.80688(0.0563)	0.71585(0.0594)	0.70391(0.0905)	0.90263(0.0612)	0.86164(0.0501)	0.86165(0.0553)
	1.3	0.80259(0.0168)	0.71232(0.0219)	0.71895(0.0213)	0.66297(0.0196)	0.68716(0.0627)	0.78255(0.0232)	0.74284(0.0168)	0.76663(0.0248)
		1.46300(0.1200)	1.16500(0.1600)	1.21900(0.1310)	1.07600(0.1430)	1.06800(0.2210)	1.38100(0.1620)	1.27800(0.1100)	1.32100(0.1370)
	1.5	0.80611(0.0175)	0.71107(0.0237)	0.72964(0.0198)	0.67168(0.0202)	0.66225(0.0602)	0.79212(0.0233)	0.74310(0.0166)	0.74466(0.0234)
		1.66969(0.1607)	1.36359(0.2210)	1.41014(0.1821)	1.24585(0.1763)	1.21534(0.2977)	1.62763(0.2276)	1.47974(0.1646)	1.48465(0.1655)
	2.50	0.78401(0.0154)	0.70868(0.0238)	0.72353(0.0191)	0.66519(0.0183)	0.67014(0.0601)	0.77370(0.0245)	0.74930(0.0174)	0.75965(0.0226)
		2.69395(0.3892)	2.22230(0.5894)	2.33646(0.4913)	2.05807(0.5098)	2.02840(0.7930)	2.62776(0.5721)	2.48233(0.4340)	2.51668(0.4297)
1.5	0.50	1.62168(0.0961)	1.41381(0.1271)	1.42016(0.1120)	1.26562(0.1052)	1.35495(0.2037)	1.61044(0.1389)	1.49207(0.0968)	1.57116(0.1462)
		0.53568(0.0106)	0.47404(0.0146)	0.47740(0.0122)	0.41767(0.0140)	0.43244(0.0187)	0.53020(0.0150)	0.49563(0.0115)	0.51292(0.0132)
	0.85	1.58041(0.0862)	1.39159(0.1201)	1.43568(0.1058)	1.29765(0.1115)	1.33374(0.2208)	1.60650(0.1430)	1.50087(0.0869)	1.54448(0.1278)
		0.90131(0.0307)	0.78432(0.0438)	0.81418(0.0372)	0.72367(0.0399)	0.73942(0.0564)	0.91379(0.0456)	0.84228(0.0322)	0.86041(0.0392)
	1.30	1.60846(0.0907)	1.38458(0.1209)	1.43594(0.1146)	1.28977(0.1046)	1.31311(0.2166)	1.57114(0.1255)	1.49823(0.0886)	1.54257(0.1286)
		1.39212(0.0735)	1.20530(0.0986)	1.24417(0.0888)	1.12098(0.0913)	1.12213(0.1303)	1.34249(0.1042)	1.30258(0.0746)	1.33718(0.0848)
	1.50	1.62091(0.0882)	1.41223(0.1237)	1.44218(0.1043)	1.29158(0.1035)	1.32121(0.2229)	1.60369(0.1356)	1.47846(0.0919)	1.54064(0.1233)
		1.60656(0.0939)	1.40372(0.1442)	1.42218(0.1139)	1.28665(0.1153)	1.29252(0.1776)	1.58817(0.1317)	1.47347(0.1013)	1.53926(0.12)
	2.50	1.61530(0.0883)	1.39348(0.1254)	1.42356(0.1111)	1.27015(0.1131)	1.33423(0.2079)	1.56833(0.1115)	1.48667(0.0967)	1.52164(0.1451)
		2.66375(0.2675)	2.32391(0.3840)	2.38408(0.3353)	2.11269(0.3573)	2.13168(0.4659)	2.63573(0.3699)	2.51503(0.2746)	2.55852(0.3441)
2	0.50	2.16103(0.1868)	1.84939(0.2540)	1.92077(0.2165)	1.70567(0.2102)	1.69743(0.3654)	2.08859(0.2469)	2.02970(0.2028)	2.01286(0.2414)
		0.53015(0.0096)	0.47071(0.0133)	0.48385(0.0115)	0.42947(0.0118)	0.42674(0.0158)	0.51594(0.0120)	0.50281(0.0094)	0.50417(0.0110)
	0.85	2.17490(0.1876)	1.83971(0.2753)	1.92606(0.2211)	1.71186(0.2213)	1.76498(0.3567)	2.16966(0.2808)	1.99158(0.1961)	2.03274(0.2611)
		0.91550(0.0260)	0.78227(0.0405)	0.82681(0.0314)	0.74106(0.0322)	0.75320(0.0470)	0.90211(0.0369)	0.84622(0.0291)	0.86478(0.0342)
	1.30	2.15764(0.1849)	1.88462(0.2495)	1.90749(0.2194)	1.68779(0.2162)	1.73314(0.3816)	2.13037(0.2490)	1.96608(0.1902)	2.08555(0.2611)
		1.39146(0.0617)	1.22292(0.0820)	1.25755(0.0776)	1.12522(0.0805)	1.13733(0.1050)	1.38473(0.0909)	1.29314(0.0739)	1.33148(0.0788)
	1.50	2.14699(0.1867)	1.87820(0.2401)	1.91775(0.2123)	1.71626(0.2048)	1.74428(0.3694)	2.16141(0.2460)	2.01294(0.1904)	2.06383(0.2805)
		1.59664(0.0789)	1.41343(0.1150)	1.44516(0.1034)	1.29742(0.1039)	1.30723(0.1446)	1.58945(0.1202)	1.51570(0.0882)	1.52888(0.1091)
	2.50	2.13181(0.1703)	1.86782(0.2231)	1.87900(0.2234)	1.71354(0.2046)	1.75912(0.4249)	2.19603(0.2626)	2.01530(0.1901)	2.09354(0.2807)
		2.64085(0.2318)	2.36851(0.3042)	2.39705(0.2884)	2.18381(0.2757)	2.16206(0.4359)	2.70630(0.3499)	2.49841(0.2529)	2.54720(0.2646)

**Table 4:** The AEs and MSEs of the MKME parameters for n=50.

*θ*	*λ*	MLEs	LSEs	WLSEs	MPSEs	PCEs	CVMEs	ADEs	RTADEs
0.25	0.50	0.25626(0.0006)	0.24640(0.0008)	0.24987(0.0006)	0.23795(0.0006)	0.23482(0.0068)	0.25326(0.0007)	0.24976(0.0006)	0.24963(0.0007)
		0.52953(0.0144)	0.46183(0.0277)	0.49293(0.0214)	0.41739(0.0197)	0.40380(0.0381)	0.51146(0.0279)	0.50000(0.0181)	0.48887(0.0177)
	0.85	0.25585(0.0006)	0.24500(0.0007)	0.24912(0.0006)	0.23849(0.0006)	0.23173(0.0060)	0.25544(0.0007)	0.25046(0.0006)	0.25466(0.0008)
		0.89423(0.0393)	0.78971(0.0681)	0.81780(0.0587)	0.72662(0.0488)	0.68771(0.1018)	0.89218(0.0733)	0.85183(0.0480)	0.87856(0.0526)
	1.30	0.25548(0.0005)	0.24562(0.0007)	0.25000(0.0006)	0.23813(0.0006)	0.23229(0.0062)	0.25449(0.0007)	0.25000(0.0005)	0.25316(0.0008)
		1.38500(0.0990)	1.19600(0.1770)	1.30100(0.1320)	1.09800(0.1190)	1.07300(0.2290)	1.35700(0.1830)	1.28500(0.1180)	1.31500(0.1180)
	1.50	0.25441(0.0005)	0.24617(0.0007)	0.24899(0.0006)	0.23865(0.0006)	0.23194(0.0058)	0.25255(0.0007)	0.25000(0.0005)	0.25065(0.0008)
		1.60004(0.1243)	1.38669(0.2222)	1.43224(0.1687)	1.27222(0.1604)	1.20938(0.3351)	1.56612(0.2482)	1.48699(0.1526)	1.51630(0.1535)
	2.50	0.25527(0.0005)	0.24892(0.0007)	0.24769(0.0005)	0.23654(0.0006)	0.23166(0.0054)	0.25495(0.0007)	0.25026(0.0006)	0.25227(0.0008)
		2.64308(0.3062)	2.43364(0.6640)	2.44557(0.4712)	2.07858(0.4476)	2.06384(0.7791)	2.61705(0.6733)	2.46905(0.4257)	2.54097(0.4245)
0.50	0.50	0.50882(0.0028)	0.49320(0.0037)	0.49833(0.0029)	0.46774(0.0030)	0.45280(0.0161)	0.51083(0.0030)	0.50021(0.0027)	0.49966(0.0035)
		0.52187(0.0090)	0.47538(0.0129)	0.49411(0.0115)	0.43638(0.0100)	0.42423(0.0206)	0.52418(0.0131)	0.50330(0.0088)	0.50260(0.0095)
	0.85	0.51148(0.0027)	0.49447(0.0037)	0.49839(0.0030)	0.47129(0.0030)	0.46575(0.0156)	0.51127(0.0035)	0.49635(0.0028)	0.50249(0.0034)
		0.89278(0.0269)	0.82925(0.0369)	0.83636(0.0319)	0.74032(0.0287)	0.72891(0.0567)	0.88529(0.0404)	0.84920(0.0275)	0.84844(0.0252)
	1.30	0.51138(0.0025)	0.49659(0.0036)	0.49478(0.0028)	0.46849(0.0029)	0.45899(0.0164)	0.51027(0.0038)	0.49874(0.0029)	0.50616(0.0037)
		1.36200(0.0590)	1.28100(0.0950)	1.25800(0.0680)	1.14500(0.0660)	1.09800(0.1410)	1.33100(0.0930)	1.28500(0.0700)	1.30100(0.0690)
	1.50	0.50899(0.0028)	0.48979(0.0036)	0.49575(0.0029)	0.47182(0.0028)	0.45487(0.0172)	0.50840(0.0037)	0.50021(0.0025)	0.50227(0.0035)
		1.55623(0.0772)	1.43526(0.1169)	1.45856(0.0907)	1.33186(0.0933)	1.27687(0.1918)	1.56720(0.1133)	1.47354(0.0864)	1.52514(0.0843)
	2.50	0.51275(0.0026)	0.48776(0.0031)	0.49605(0.0028)	0.46914(0.0030)	0.45983(0.0171)	0.50550(0.0030)	0.50063(0.0029)	0.50381(0.0036)
		2.60041(0.2078)	2.39927(0.3240)	2.43802(0.2286)	2.17744(0.2816)	2.10254(0.4987)	2.58517(0.2973)	2.50121(0.2366)	2.47866(0.2259)
0.75	0.5	0.76913(0.0063)	0.74205(0.0089)	0.74494(0.0077)	0.69558(0.0078)	0.68063(0.0333)	0.76213(0.0096)	0.74390(0.0073)	0.75536(0.0092)
		0.51786(0.0067)	0.47813(0.0091)	0.48956(0.0076)	0.45021(0.0076)	0.43606(0.0143)	0.50719(0.0089)	0.49617(0.0068)	0.49845(0.0077)
	0.85	0.76204(0.0065)	0.73234(0.0090)	0.74218(0.0078)	0.70117(0.0074)	0.69033(0.0311)	0.76317(0.0089)	0.75025(0.0070)	0.75496(0.0094)
		0.88584(0.0186)	0.82562(0.0258)	0.82867(0.0226)	0.76423(0.0217)	0.74082(0.0400)	0.87777(0.0256)	0.84537(0.0204)	0.85929(0.0227)
	1.3	0.76319(0.0064)	0.73804(0.0080)	0.74814(0.0077)	0.69739(0.0078)	0.68858(0.0308)	0.76676(0.0089)	0.75181(0.0067)	0.75070(0.0085)
		1.35200(0.0430)	1.26100(0.0580)	1.29100(0.0470)	1.16300(0.0490)	1.14800(0.0970)	1.34400(0.0620)	1.29400(0.0480)	1.30400(0.047)
	1.5	0.76347(0.0061)	0.73394(0.0089)	0.74350(0.0072)	0.69950(0.0077)	0.67956(0.0343)	0.76445(0.0098)	0.74858(0.0066)	0.76096(0.0089)
		1.54834(0.0537)	1.44594(0.0819)	1.48299(0.0625)	1.34911(0.0679)	1.29296(0.1286)	1.52879(0.0844)	1.49654(0.0636)	1.51824(0.0623)
	2.50	0.77074(0.0062)	0.73300(0.0096)	0.74384(0.0076)	0.69940(0.0079)	0.68824(0.0319)	0.77154(0.0093)	0.74994(0.0068)	0.75802(0.0096)
		2.59367(0.1518)	2.40310(0.2337)	2.46813(0.1837)	2.24047(0.2096)	2.18059(0.3481)	2.60709(0.2488)	2.46930(0.1799)	2.50672(0.1876)
1.5	0.50	1.55810(0.0392)	1.46250(0.0515)	1.47833(0.0413)	1.38147(0.0415)	1.34270(0.1075)	1.52918(0.0494)	1.48740(0.0374)	1.51182(0.0559)
		0.51812(0.0040)	0.48230(0.0062)	0.49052(0.0049)	0.45601(0.0052)	0.44803(0.0090)	0.50727(0.0060)	0.49288(0.0046)	0.49742(0.0049)
	0.85	1.54544(0.0364)	1.45733(0.0498)	1.47907(0.0410)	1.38075(0.0425)	1.36545(0.1026)	1.52042(0.0507)	1.49184(0.0392)	1.51356(0.0485)
		0.87033(0.0116)	0.82542(0.0181)	0.84146(0.0134)	0.78156(0.0153)	0.77269(0.0276)	0.85984(0.0171)	0.84814(0.0135)	0.85518(0.0133)
	1.30	1.55646(0.0371)	1.45497(0.0462)	1.47382(0.0388)	1.38241(0.0380)	1.34025(0.1072)	1.53359(0.0483)	1.50162(0.0339)	1.51691(0.0540)
		1.34188(0.0319)	1.26166(0.0380)	1.27702(0.0322)	1.19537(0.0326)	1.17020(0.0547)	1.33196(0.0394)	1.31137(0.0299)	1.31588(0.0366)
	1.50	1.53995(0.0313)	1.45734(0.0517)	1.48757(0.0380)	1.37663(0.0431)	1.35365(0.1147)	1.53686(0.0461)	1.50332(0.0407)	1.52169(0.0485)
		1.54047(0.0397)	1.44484(0.0607)	1.48662(0.0389)	1.37354(0.0494)	1.35410(0.0821)	1.53327(0.0577)	1.50624(0.0429)	1.50915(0.0454)
	2.50	1.53969(0.0325)	1.46104(0.0462)	1.47695(0.0427)	1.37354(0.0394)	1.35159(0.1118)	1.53257(0.0485)	1.50090(0.0373)	1.52241(0.0526)
		2.56563(0.1033)	2.43469(0.1456)	2.46970(0.1262)	2.27536(0.1305)	2.25437(0.2057)	2.54205(0.1341)	2.49017(0.1096)	2.53076(0.1362)
2	0.50	2.06982(0.0672)	1.96808(0.0925)	1.96454(0.0796)	1.80851(0.0886)	1.80980(0.1934)	2.04374(0.0945)	1.98387(0.0709)	2.00930(0.1032)
		0.51535(0.0032)	0.49228(0.0050)	0.49658(0.0039)	0.45850(0.0044)	0.45830(0.0070)	0.50603(0.0047)	0.50013(0.0041)	0.49770(0.0042)
	0.85	2.05106(0.0686)	1.94400(0.1119)	1.96939(0.0786)	1.82869(0.0820)	1.82539(0.1878)	2.05443(0.1058)	2.01260(0.0741)	2.02320(0.1195)
		0.86649(0.0103)	0.82959(0.0151)	0.84316(0.0131)	0.79072(0.0115)	0.78382(0.0202)	0.86593(0.0149)	0.84914(0.0106)	0.84755(0.0127)
	1.30	2.07279(0.0717)	1.95007(0.0944)	1.97681(0.0800)	1.82691(0.0826)	1.80495(0.1818)	2.04826(0.0967)	2.00317(0.0733)	2.01597(0.1050)
		1.34617(0.0246)	1.26471(0.0321)	1.29360(0.0279)	1.19188(0.0298)	1.18788(0.0482)	1.32682(0.0313)	1.30215(0.0269)	1.30292(0.0331)
	1.50	2.05527(0.0698)	1.95131(0.1000)	1.98136(0.0772)	1.80909(0.0903)	1.79760(0.1989)	2.04658(0.0999)	2.01173(0.0777)	2.00542(0.1027)
		1.53343(0.0319)	1.47265(0.0429)	1.49505(0.0373)	1.36995(0.0422)	1.36661(0.0713)	1.52917(0.0448)	1.49578(0.0368)	1.49858(0.0391)
	2.50	2.06316(0.0720)	1.93476(0.1043)	1.95593(0.0837)	1.83059(0.0816)	1.80950(0.1801)	2.02351(0.1055)	1.98412(0.0817)	2.02830(0.1116)
		2.56994(0.0915)	2.43965(0.1206)	2.47717(0.1061)	2.32069(0.1082)	2.28582(0.1812)	2.53029(0.1223)	2.48802(0.1019)	2.52307(0.1229)

**Table 5:** The AEs and MSEs of the MKME parameters for n=100.

*θ*	*λ*	MLEs	LSEs	WLSEs	MPSEs	PCEs	CVMEs	ADEs	RTADEs
0.25	0.50	0.25329(0.0003)	0.24852(0.0004)	0.24938(0.0003)	0.24227(0.0003)	0.23035(0.0037)	0.25216(0.0004)	0.25000(0.0003)	0.24892(0.0004)
		0.52446(0.0081)	0.47975(0.0132)	0.49199(0.0104)	0.44303(0.0088)	0.42582(0.0197)	0.51446(0.0143)	0.50000(0.0093)	0.5079(0.0097)
	0.85	0.25206(0.0002)	0.24789(0.0003)	0.24994(0.0003)	0.24244(0.0003)	0.23127(0.0037)	0.25235(0.0003)	0.25000(0.0003)	0.2508(0.0003)
		0.86962(0.0210)	0.82319(0.0378)	0.83868(0.0288)	0.76228(0.0228)	0.72782(0.0548)	0.86005(0.0387)	0.85000(0.0269)	0.86159(0.0254)
	1.30	0.25159(0.0003)	0.24895(0.0003)	0.24929(0.0003)	0.24206(0.0003)	0.23031(0.0037)	0.25293(0.0003)	0.25000(0.0002)	0.24956(0.0003)
		1.33000(0.0440)	1.24700(0.0830)	1.28300(0.0690)	1.16800(0.0580)	1.10100(0.1290)	1.32900(0.0880)	1.29600(0.0550)	1.29900(0.0560)
	1.50	0.25271(0.0003)	0.24832(0.0004)	0.25087(0.0003)	0.24302(0.0003)	0.23284(0.0037)	0.25265(0.0004)	0.25054(0.0003)	0.24946(0.0003)
		1.55811(0.0524)	1.43842(0.1043)	1.47544(0.0869)	1.35843(0.0744)	1.30106(0.1611)	1.51722(0.1256)	1.50000(0.0782)	1.48314(0.0716)
	2.50	0.25147(0.0003)	0.24839(0.0004)	0.24962(0.0003)	0.24256(0.0003)	0.23438(0.0036)	0.25380(0.0003)	0.24992(0.0003)	0.24992(0.0003)
		2.56992(0.1544)	2.40042(0.3773)	2.45974(0.2414)	2.26488(0.2005)	2.14820(0.4852)	2.55021(0.3325)	2.50000(0.2250)	2.51837(0.2119)
0.50	0.50	0.50514(0.0012)	0.49456(0.0016)	0.49918(0.0015)	0.48308(0.0014)	0.46371(0.0098)	0.50264(0.0019)	0.49896(0.0013)	0.50153(0.0018)
		0.51269(0.0043)	0.49446(0.0060)	0.49648(0.0052)	0.46335(0.0049)	0.44403(0.0102)	0.50779(0.0063)	0.49766(0.0046)	0.50072(0.0049)
	0.85	0.50784(0.0014)	0.49415(0.0018)	0.50004(0.0014)	0.47879(0.0015)	0.46509(0.0107)	0.50338(0.0016)	0.50067(0.0013)	0.50161(0.0018)
		0.87685(0.0126)	0.83436(0.0205)	0.84480(0.0149)	0.78736(0.0146)	0.76260(0.0321)	0.86305(0.0189)	0.84554(0.0136)	0.85255(0.0144)
	1.30	0.50538(0.0012)	0.49523(0.0017)	0.49939(0.0015)	0.47796(0.0014)	0.46477(0.0108)	0.50727(0.0018)	0.49909(0.0014)	0.49962(0.0019)
		1.32100(0.0310)	1.26800(0.0440)	1.28900(0.0380)	1.21000(0.0350)	1.15500(0.0760)	1.32000(0.0450)	1.28200(0.0320)	1.29100(0.0330)
	1.50	0.5054(0.0014)	0.49562(0.0015)	0.49878(0.0014)	0.48320(0.0014)	0.46712(0.0093)	0.50508(0.0017)	0.50223(0.0014)	0.50365(0.0017)
		1.5311(0.0394)	1.46358(0.0618)	1.48846(0.0480)	1.39572(0.0462)	1.36658(0.0894)	1.51623(0.0525)	1.50202(0.0447)	1.49480(0.0453)
	2.50	0.50576(0.0014)	0.4978(0.0015)	0.50027(0.0013)	0.48117(0.0014)	0.46386(0.0097)	0.50244(0.0016)	0.49953(0.0012)	0.50287(0.0018)
		2.53728(0.1087)	2.4297(0.1432)	2.46892(0.1256)	2.31549(0.1184)	2.25909(0.2666)	2.55140(0.1507)	2.49293(0.1220)	2.52920(0.1363)
0.75	0.5	0.75907(0.0034)	0.74445(0.0042)	0.74780(0.0038)	0.71811(0.0035)	0.70200(0.0176)	0.75498(0.0046)	0.74840(0.0039)	0.75633(0.0044)
		0.51083(0.0032)	0.49332(0.0049)	0.49403(0.0037)	0.46449(0.0037)	0.45761(0.0077)	0.50782(0.0045)	0.49957(0.0039)	0.49884(0.0041)
	0.85	0.76082(0.0034)	0.74048(0.0044)	0.74866(0.0038)	0.72019(0.0035)	0.69386(0.0190)	0.75855(0.0042)	0.74938(0.0034)	0.74850(0.0051)
		0.86967(0.0091)	0.83355(0.0128)	0.83673(0.0109)	0.79246(0.0104)	0.77525(0.0223)	0.86425(0.0128)	0.86094(0.0106)	0.84759(0.0116)
	1.3	0.76151(0.0032)	0.74152(0.0048)	0.75141(0.0033)	0.72039(0.0033)	0.69647(0.0198)	0.75582(0.0043)	0.75132(0.0034)	0.74969(0.0047)
		1.33600(0.0230)	1.28000(0.032)0	1.29900(0.0220)	1.22200(0.0250)	1.19100(0.052)0	1.32000(0.0300)	1.30500(0.0240)	1.30000(0.0260)
	1.5	0.75934(0.0034)	0.74091(0.0044)	0.74979(0.0036)	0.71845(0.0034)	0.6957(0.0185)	0.7573(0.0049)	0.75263(0.0037)	0.75311(0.0047)
		1.53106(0.0319)	1.48044(0.0400)	1.49341(0.0349)	1.40484(0.0275)	1.37864(0.066)	1.51496(0.041)	1.48303(0.0324)	1.50719(0.0312)
	2.50	0.76155(0.0034)	0.74142(0.0040)	0.74708(0.0042)	0.72080(0.0038)	0.69011(0.0194)	0.75815(0.0045)	0.75244(0.0036)	0.75012(0.0050)
		2.55858(0.0799)	2.43701(0.1118)	2.47490(0.0852)	2.34145(0.0985)	2.26469(0.2095)	2.53851(0.1206)	2.49951(0.0929)	2.50487(0.0928)
1.5	0.50	1.51960(0.0158)	1.47309(0.0249)	1.49134(0.0201)	1.43466(0.0195)	1.37111(0.0639)	1.51731(0.0248)	1.49643(0.0176)	1.49683(0.0269)
		0.50390(0.0022)	0.49354(0.0032)	0.49828(0.0024)	0.47795(0.0024)	0.46492(0.0045)	0.50443(0.0027)	0.49820(0.0022)	0.50169(0.0029)
	0.85	1.51535(0.0161)	1.48946(0.0244)	1.49426(0.0193)	1.41766(0.0215)	1.38316(0.0640)	1.52028(0.0251)	1.50463(0.0194)	1.51242(0.0265)
		0.85881(0.0059)	0.84402(0.0080)	0.84582(0.0067)	0.80509(0.0073)	0.78900(0.0139)	0.86527(0.0083)	0.85591(0.0066)	0.85445(0.0075)
	1.30	1.51761(0.0180)	1.46979(0.0248)	1.49746(0.0190)	1.41957(0.0191)	1.39797(0.0644)	1.50473(0.0246)	1.49618(0.0181)	1.49847(0.0245)
		1.31400(0.0137)	1.26746(0.0183)	1.29752(0.0163)	1.22906(0.0157)	1.21579(0.0306)	1.31608(0.0197)	1.30108(0.0147)	1.30395(0.0187)
	1.50	1.51717(0.0153)	1.46407(0.0249)	1.49461(0.0185)	1.42357(0.0180)	1.38527(0.0582)	1.53311(0.0239)	1.49414(0.0176)	1.49475(0.0259)
		1.53388(0.0185)	1.46436(0.0242)	1.49505(0.0214)	1.42015(0.0205)	1.40538(0.0390)	1.51939(0.0244)	1.49910(0.0209)	1.50252(0.0241)
	2.50	1.53606(0.0168)	1.48882(0.0260)	1.48856(0.0205)	1.41434(0.0204)	1.37825(0.0627)	1.52490(0.0235)	1.49661(0.0175)	1.50150(0.0256)
		2.52897(0.0552)	2.47522(0.0727)	2.48651(0.0614)	2.35883(0.0626)	2.31834(0.1115)	2.54009(0.0711)	2.49088(0.0568)	2.49105(0.0614)
2	0.50	2.02925(0.0371)	1.96514(0.0467)	1.99553(0.0389)	1.90372(0.0383)	1.84775(0.1021)	2.03043(0.0499)	1.98982(0.0346)	1.99319(0.0488)
		0.50622(0.0020)	0.49258(0.0025)	0.49858(0.0020)	0.47901(0.0017)	0.47210(0.0039)	0.50723(0.0028)	0.49677(0.0020)	0.49688(0.0024)
	0.85	2.01310(0.0353)	1.97167(0.0522)	2.00115(0.0412)	1.89890(0.0401)	1.85671(0.1119)	2.03508(0.0531)	1.98083(0.0386)	2.01912(0.0525)
		0.86153(0.0051)	0.84165(0.0075)	0.84908(0.0058)	0.80806(0.0058)	0.80342(0.0097)	0.86487(0.0075)	0.84712(0.0055)	0.85710(0.0066)
	1.30	2.03559(0.0337)	1.95191(0.0498)	1.99612(0.0387)	1.88467(0.0382)	1.84798(0.1122)	2.02384(0.0545)	1.99810(0.0418)	1.99880(0.0544)
		1.31658(0.0117)	1.27861(0.0180)	1.29919(0.0127)	1.23235(0.0143)	1.22538(0.0251)	1.30630(0.0164)	1.29841(0.0135)	1.29967(0.0165)
	1.50	2.02907(0.0334)	1.97325(0.0443)	1.99376(0.0421)	1.88296(0.0411)	1.87600(0.1094)	2.01652(0.0488)	2.00820(0.0346)	2.02802(0.0579)
		1.52445(0.0156)	1.48115(0.0217)	1.49576(0.0208)	1.42484(0.0191)	1.42478(0.0337)	1.52186(0.0218)	1.50088(0.0178)	1.50995(0.0214)
	2.50	2.01830(0.0331)	1.97488(0.0532)	1.98493(0.0453)	1.89018(0.0407)	1.84824(0.1136)	2.02564(0.0486)	2.00124(0.0417)	1.99568(0.0563)
		2.51285(0.0419)	2.47746(0.0670)	2.49197(0.0540)	2.38571(0.0492)	2.34110(0.0903)	2.52144(0.0614)	2.50625(0.0490)	2.49516(0.0553)

**Table 6:** The AEs and MSEs of the MKME parameters for n=250.

*θ*	*λ*	MLEs	LSEs	WLSEs	MPSEs	PCEs	CVMEs	ADEs	RTADEs
0.25	0.50	0.25136(0.0001)	0.24949(0.0001)	0.24986(0.0001)	0.24706(0.0001)	0.23441(0.0018)	0.25119(0.0001)	0.25000(0.0001)	0.24986(0.0001)
		0.50351(0.0027)	0.49527(0.0053)	0.4992(0.0038)	0.47721(0.0030)	0.45126(0.0082)	0.50638(0.0058)	0.50000(0.0039)	0.49727(0.0036)
	0.85	0.25126(0.0001)	0.24895(0.0001)	0.25121(0.0001)	0.24726(0.0001)	0.23436(0.0018)	0.25043(0.0001)	0.25000(0.0001)	0.25070(0.0002)
		0.85800(0.0069)	0.83409(0.0163)	0.85206(0.0114)	0.81413(0.0084)	0.76852(0.0241)	0.85737(0.0157)	0.85000(0.0095)	0.85417(0.0101)
	1.30	0.25056(0.0001)	0.24928(0.0001)	0.25088(0.0001)	0.24672(0.0001)	0.23216(0.0018)	0.25001(0.0001)	0.25000(0.0001)	0.25039(0.0001)
		1.30900(0.0160)	1.27900(0.0350)	1.30400(0.0250)	1.23600(0.0210)	1.17200(0.0570)	1.31800(0.0380)	1.30000(0.0230)	1.29900(0.0250)
	1.50	0.25063(0.0001)	0.24914(0.0001)	0.25058(0.0001)	0.24695(0.0001)	0.23462(0.0018)	0.25070(0.0001)	0.25000(0.0001)	0.24949(0.0001)
		1.51005(0.0212)	1.48719(0.0451)	1.48328(0.0320)	1.44023(0.0245)	1.36400(0.0729)	1.50999(0.0509)	1.50000(0.0303)	1.48576(0.0281)
	2.50	0.25063(0.0001)	0.25004(0.0001)	0.24972(0.0001)	0.24708(0.0001)	0.23397(0.0018)	0.25118(0.0001)	0.25000(0.0001)	0.25047(0.0001)
		2.51657(0.0578)	2.48743(0.1338)	2.47794(0.0953)	2.49454(0.0668)	2.27128(0.2198)	2.50637(0.1278)	2.50000(0.0880)	2.50160(0.0702)
0.50	0.50	0.50128(0.0005)	0.4986(0.0006)	0.49987(0.0006)	0.49046(0.0005)	0.46943(0.0047)	0.50179(0.0007)	0.50052(0.0006)	0.50146(0.0007)
		0.50359(0.0016)	0.49736(0.0026)	0.50156(0.0021)	0.47957(0.0018)	0.46635(0.0043)	0.50151(0.0026)	0.49601(0.0020)	0.50223(0.0021)
	0.85	0.5023(0.0005)	0.49776(0.0007)	0.49932(0.0005)	0.49070(0.0005)	0.46928(0.0046)	0.50080(0.0007)	0.49966(0.0005)	0.50044(0.0007)
		0.85854(0.0048)	0.84775(0.0077)	0.85042(0.0058)	0.81589(0.0049)	0.79225(0.0130)	0.85850(0.0074)	0.84786(0.0054)	0.84568(0.0058)
	1.30	0.50267(0.0005)	0.49705(0.0007)	0.49999(0.0006)	0.48933(0.0005)	0.46743(0.0049)	0.50209(0.0007)	0.49868(0.0006)	0.49990(0.0008)
		1.31500(0.0110)	1.28800(0.0180)	1.31000(0.0140)	1.24600(0.0110)	1.20200(0.0310)	1.31000(0.0170)	1.29300(0.0140)	1.29100(0.0130)
	1.50	0.50057(0.0005)	0.49814(0.0007)	0.50054(0.0006)	0.49122(0.0006)	0.47058(0.0050)	0.50214(0.0007)	0.49831(0.0006)	0.50011(0.0007)
		1.50517(0.0139)	1.48389(0.0253)	1.49485(0.0189)	1.44734(0.0160)	1.38761(0.0380)	1.51355(0.0234)	1.49264(0.0170)	1.50118(0.0173)
	2.50	0.50121(0.0005)	0.49871(0.0006)	0.49931(0.0005)	0.49064(0.0005)	0.47274(0.0050)	0.50184(0.0007)	0.49860(0.0005)	0.50086(0.0007)
		2.52826(0.0407)	2.47642(0.0639)	2.50383(0.0471)	2.39296(0.0484)	2.33184(0.1084)	2.52844(0.0567)	2.50333(0.0496)	2.49659(0.0500)
0.75	0.5	0.75272(0.0013)	0.74706(0.0017)	0.74919(0.0014)	0.73431(0.0013)	0.70693(0.0087)	0.75375(0.0019)	0.74795(0.0014)	0.75326(0.0019)
		0.50425(0.0015)	0.49741(0.0018)	0.49917(0.0014)	0.48401(0.0014)	0.46952(0.0034)	0.50659(0.0019)	0.50127(0.0014)	0.49897(0.0015)
	0.85	0.75175(0.0012)	0.75097(0.0018)	0.75000(0.0013)	0.73647(0.0013)	0.71187(0.0080)	0.75321(0.0017)	0.75246(0.0015)	0.75021(0.0016)
		0.85706(0.0034)	0.84501(0.0055)	0.84513(0.0046)	0.82628(0.0038)	0.79984(0.0092)	0.85458(0.0053)	0.84949(0.0040)	0.85156(0.0042)
	1.3	0.75229(0.0012)	0.74994(0.0018)	0.75291(0.0014)	0.73653(0.0014)	0.70830(0.0091)	0.75439(0.0016)	0.74941(0.0014)	0.75291(0.0018)
		1.31000(0.0080)	1.30200(0.0130)	1.30600(0.0100)	1.25800(0.0100)	1.22100(0.0210)	1.30900(0.0120)	1.30200(0.0090)	1.30300(0.0100)
	1.5	0.75404(0.0013)	0.74464(0.0018)	0.74943(0.0015)	0.73405(0.0014)	0.71832(0.0073)	0.75132(0.0019)	0.74997(0.0013)	0.75066(0.0018)
		1.51116(0.0125)	1.47850(0.0168)	1.49620(0.0145)	1.45532(0.0123)	1.42108(0.0262)	1.51304(0.0176)	1.50451(0.0134)	1.50558(0.0145)
	2.50	0.75193(0.0012)	0.74534(0.0018)	0.75301(0.0016)	0.73375(0.0014)	0.70458(0.0092)	0.75502(0.0016)	0.7487(0.0015)	0.74843(0.0019)
		2.51204(0.0299)	2.47945(0.0462)	2.50612(0.0390)	2.42354(0.0339)	2.34165(0.0837)	2.50557(0.0453)	2.5028(0.0336)	2.50514(0.0395)
1.5	0.50	1.51165(0.0065)	1.48393(0.0099)	1.50253(0.0078)	1.46082(0.0073)	1.42041(0.0295)	1.50544(0.0093)	1.49823(0.0071)	1.50264(0.0106)
		0.50236(0.0008)	0.49568(0.0011)	0.50286(0.0009)	0.48828(0.0009)	0.47937(0.0019)	0.50219(0.0011)	0.49999(0.0009)	0.50062(0.0011)
	0.85	1.51192(0.0070)	1.49123(0.0100)	1.4983(0.00730)	1.46314(0.0079)	1.42703(0.0269)	1.50627(0.0090)	1.50097(0.0078)	1.50511(0.0109)
		0.85330(0.0023)	0.84093(0.0035)	0.84962(0.0027)	0.82632(0.0027)	0.81717(0.0055)	0.85519(0.0031)	0.84974(0.0025)	0.85517(0.0028)
	1.30	1.50745(0.0063)	1.49438(0.0094)	1.49691(0.0075)	1.46010(0.0076)	1.43683(0.0262)	1.50150(0.0094)	1.49361(0.0078)	1.49949(0.0105)
		1.30812(0.0052)	1.30073(0.0079)	1.29994(0.0066)	1.26624(0.0062)	1.25178(0.0116)	1.30433(0.0076)	1.29736(0.0064)	1.30385(0.0069)
	1.50	1.51274(0.0067)	1.49882(0.0100)	1.49936(0.0080)	1.46260(0.0072)	1.43539(0.0292)	1.51935(0.0101)	1.49814(0.0078)	1.50611(0.0112)
		1.51176(0.0068)	1.49727(0.0103)	1.49922(0.0087)	1.45671(0.0079)	1.44920(0.0185)	1.51538(0.0098)	1.49587(0.0078)	1.50513(0.0096)
	2.50	1.50808(0.0061)	1.48959(0.0091)	1.49460(0.0078)	1.46155(0.0071)	1.43312(0.0284)	1.50409(0.0094)	1.49820(0.0075)	1.50186(0.0110)
		2.50308(0.0196)	2.48064(0.0277)	2.49902(0.0236)	2.43699(0.0229)	2.40599(0.0476)	2.51883(0.0302)	2.49806(0.0229)	2.50543(0.0256)
2	0.50	2.01538(0.0154)	1.98212(0.0206)	2.00119(0.0164)	1.94589(0.0156)	1.90836(0.0461)	2.01590(0.0197)	1.99694(0.0161)	2.00213(0.0210)
		0.50347(0.0007)	0.49597(0.0011)	0.49903(0.0008)	0.48794(0.0008)	0.48381(0.0015)	0.50291(0.0010)	0.49992(0.0008)	0.50009(0.0008)
	0.85	2.01733(0.0142)	1.98746(0.0182)	2.00166(0.0144)	1.93615(0.0152)	1.89811(0.0485)	2.00289(0.0182)	2.00202(0.0167)	2.00456(0.0206)
		0.85727(0.0022)	0.84824(0.0028)	0.85069(0.0021)	0.82839(0.0022)	0.81619(0.0046)	0.85050(0.0028)	0.85374(0.0023)	0.85117(0.0027)
	1.30	2.00704(0.0143)	1.99343(0.0196)	2.00629(0.0153)	1.93873(0.0162)	1.90239(0.0516)	2.01439(0.0208)	1.99372(0.0180)	2.00013(0.0213)
		1.30504(0.0044)	1.28875(0.0065)	1.30320(0.0054)	1.26639(0.0054)	1.24750(0.0109)	1.30897(0.0070)	1.30081(0.0057)	1.30378(0.0057)
	1.50	2.02399(0.0127)	2.00148(0.0206)	2.00529(0.0155)	1.94036(0.0164)	1.89457(0.0485)	2.00378(0.0190)	2.00448(0.0157)	2.00276(0.0200)
		1.50997(0.0062)	1.50029(0.0092)	1.49585(0.0080)	1.46281(0.0076)	1.44122(0.0147)	1.50328(0.0087)	1.50091(0.0072)	1.50318(0.0082)
	2.50	2.01449(0.0152)	1.98847(0.0194)	1.99858(0.0151)	1.94633(0.0154)	1.90520(0.0494)	2.01126(0.0198)	1.99354(0.0148)	2.00778(0.0226)
		2.51970(0.0169)	2.49687(0.0245)	2.48570(0.0185)	2.44222(0.0186)	2.41152(0.0394)	2.51470(0.0240)	2.49562(0.0199)	2.50968(0.0239)

## 7 Lifetime and count data analysis in applied sciences

This section is devoted to showing the empirical flexibility of the special models of the two proposed families using some real-life data, including both continuous and discrete data.

### 7.1 Applications for the MKME model

In this subsection, we analyze five real-life datasets from applied fields including environmental sciences, medicine, and engineering to explore the flexibility of the MKME model. The first dataset refers to waiting times (in minutes) before the service of 100 bank customers. This dataset is studied by [24]. The data observations are: 3.2, 0.8, 4.6, 1.9, 0.8, 3.3, 6.2, 4.7, 6.2, 8, 7.7, 9.7, 12.5, 9.8, 12.9, 18.1, 17.3, 27, 1.3, 31.6, 3.5, 6.2, 4.7, 8.2, 13, 10.7, 18.2, 13, 1.5, 33.1, 1.8, 3.6, 1.9, 4, 2.6, 4.8, 4.1, 4.9, 6.3, 4.9, 6.7, 4.2, 8.6, 11, 6.9, 8.6, 11.2, 10.9, 8.6, 11, 13.7, 11.2, 13.6, 13.3, 18.4, 19, 18.9, 38.5, 2.1, 4.2, 5, 4.3, 5.3, 7.1, 5.5, 7.1, 8.8, 7.1, 8.9, 11.1, 8.8, 13.9, 19.9, 14.1, 20.6, 2.7, 21.3, 4.4, 2.9, 4.3, 3.1, 21.9, 4.4, 5.7, 9.6, 7.1, 6.1, 7.4, 8.9, 7.6, 5.7, 11.5, 9.5, 11.9, 15.4, 12.4, 17.3, 15.4, 23, 21.4.

The second dataset is discussed [25], and it contains annual maximum flood levels over a 20-year period of the Susquehanna River at Harrisburg, Pennsylvania. These flood levels are measured in millions cubic of feet per second. The data observations are: 0.494, 0.654, 0.315, 0.297, 0.449, 0.379, 0.402, 0.379, 0.423, 0.324, 0.740, 0.269, 0.418, 0.416, 0.412, 0.338, 0.484, 0.392, 0.265, 0.613.

The third dataset is analyzed by [26], and it refers to remission times (in months) for 128 bladder cancer patients. This dataset contains the following observations: 3.48, 0.08, 2.09, 4.87, 8.66, 6.94, 13.11, 0.20, 23.63, 2.23, 4.98, 3.52, 6.97, 13.29, 9.02, 0.40, 3.57, 2.26, 5.06, 9.22, 7.09, 13.80, 0.50, 25.74, 2.46, 5.09, 3.64, 7.26, 14.24, 9.47, 25.82, 2.54, 0.51, 3.70, 7.28, 5.17, 9.74, 26.31, 14.76, 0.81, 3.82, 2.62, 5.32, 10.06, 7.32, 14.77, 2.64, 32.15, 3.88, 7.39, 5.32, 10.34, 34.26, 14.83, 0.90, 4.18, 2.69, 5.34, 10.66, 7.59, 15.96, 1.05, 36.66, 2.69, 5.41, 4.23, 7.62, 16.62, 10.75, 43.01, 2.75, 1.19, 4.26, 7.63, 5.41, 17.12, 12.63, 1.26, 46.12, 2.83, 5.49, 4.33, 7.66, 17.14, 11.25, 79.05, 2.87, 1.35, 5.62, 11.64, 7.87, 17.36, 3.02, 1.40, 4.34, 7.93, 5.71, 11.79, 1.46, 18.10, 4.40, 8.26, 5.85, 11.98, 1.76, 19.13, 3.25, 6.25, 4.50, 8.37, 2.02, 22.69, 12.02, 3.31, 6.54, 4.51, 8.53, 20.28, 12.03, 2.02, 6.76, 3.36, 12.07, 2.07, 21.73, 3.36, 8.65, 6.93.

The fourth dataset is studied by [27], and it refers to the number of vehicle fatalities for 39 counties in South Carolina in the year 2012. The data observations are: 50, 22, 13, 17, 26, 4, 9, 27, 9, 48, 31, 20, 6, 12, 5, 9, 14, 16, 33, 9, 68, 3, 20, 51, 4, 13, 17, 2, 16, 52, 6, 48, 12, 23, 10, 8, 13, 1, 15.

The fifth dataset is considered by [28]. This dataset refers to the time between failures for 30 repairable items, and its observations are: 0.70, 0.11, 1.43, 0.71, 2.63, 0.77, 1.49, 2.46, 3.46, 0.59, 1.23, 0.74, 1.17, 0.94, 0.40, 4.36, 1.74, 2.23, 4.73, 0.45, 1.46, 1.06, 0.30, 2.37, 1.82, 0.63, 1.24, 1.23, 1.86, 1.97.

The ML method is used to estimate the model parameters from each dataset, and the R program is used to obtain different computations. The fitting performance of the MKME distribution is compared to other competing E models including the generalized E (GE) [29], Marshall–Olkin E (MOE), alpha-power E (APE) [30], generalized DUS E (GDUSE) [31], generalized inverted E (GIE) [32], KME, and E distributions.

The fitting performance of the proposed MKME model and other competing distributions is explored using some goodness-of-fit measures including the Anderson–Darling $\left(A^{*}\right),$ Cramér–von Mises $\left(W^{*}\right),$ and Kolmogorov–Smirnov (KS) statistics with its associated *p*-value.

**Fig 8:**
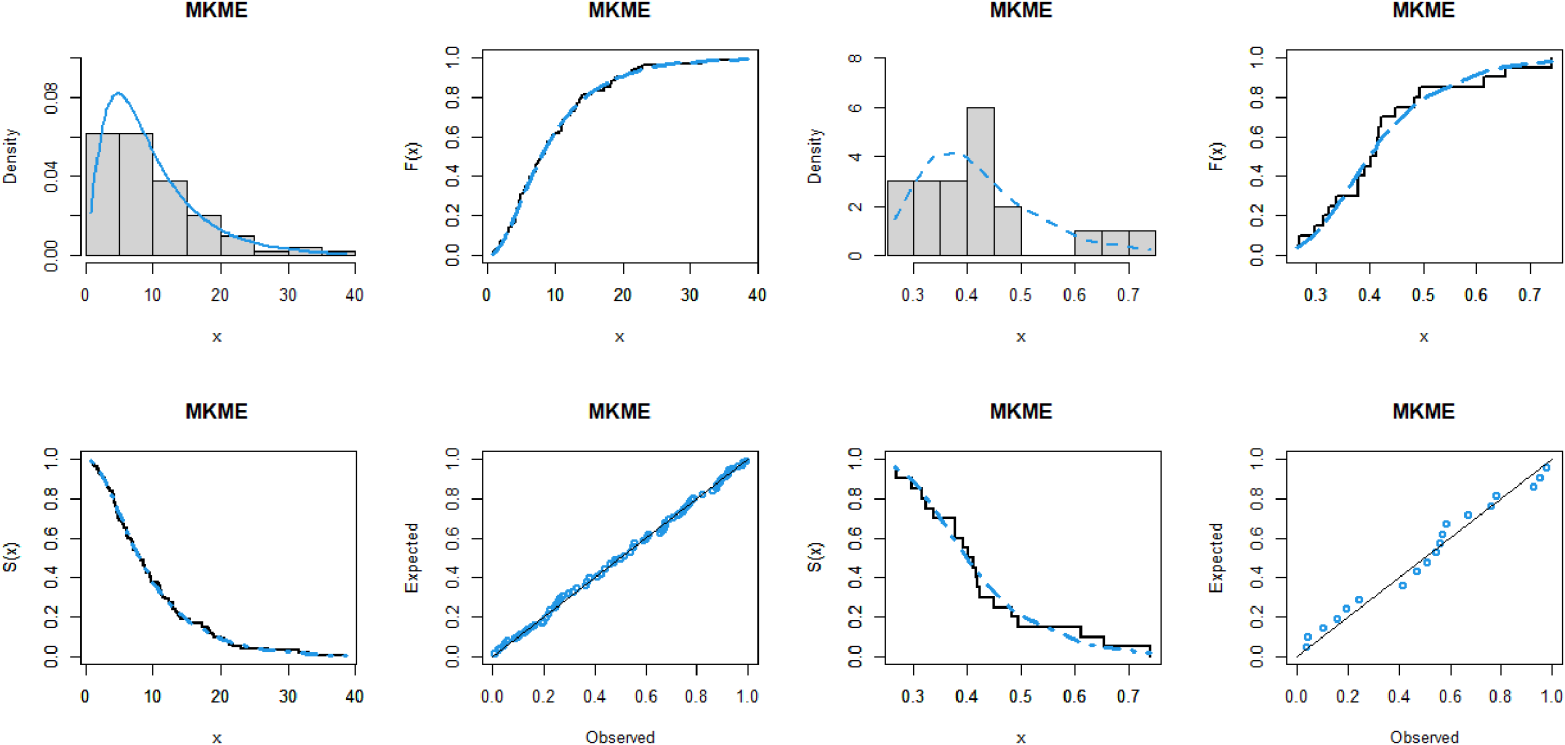
The fitted functions plots of the MKME model for waiting times dataset (left panel) and flood levels dataset (right panel).

**Fig 9:**
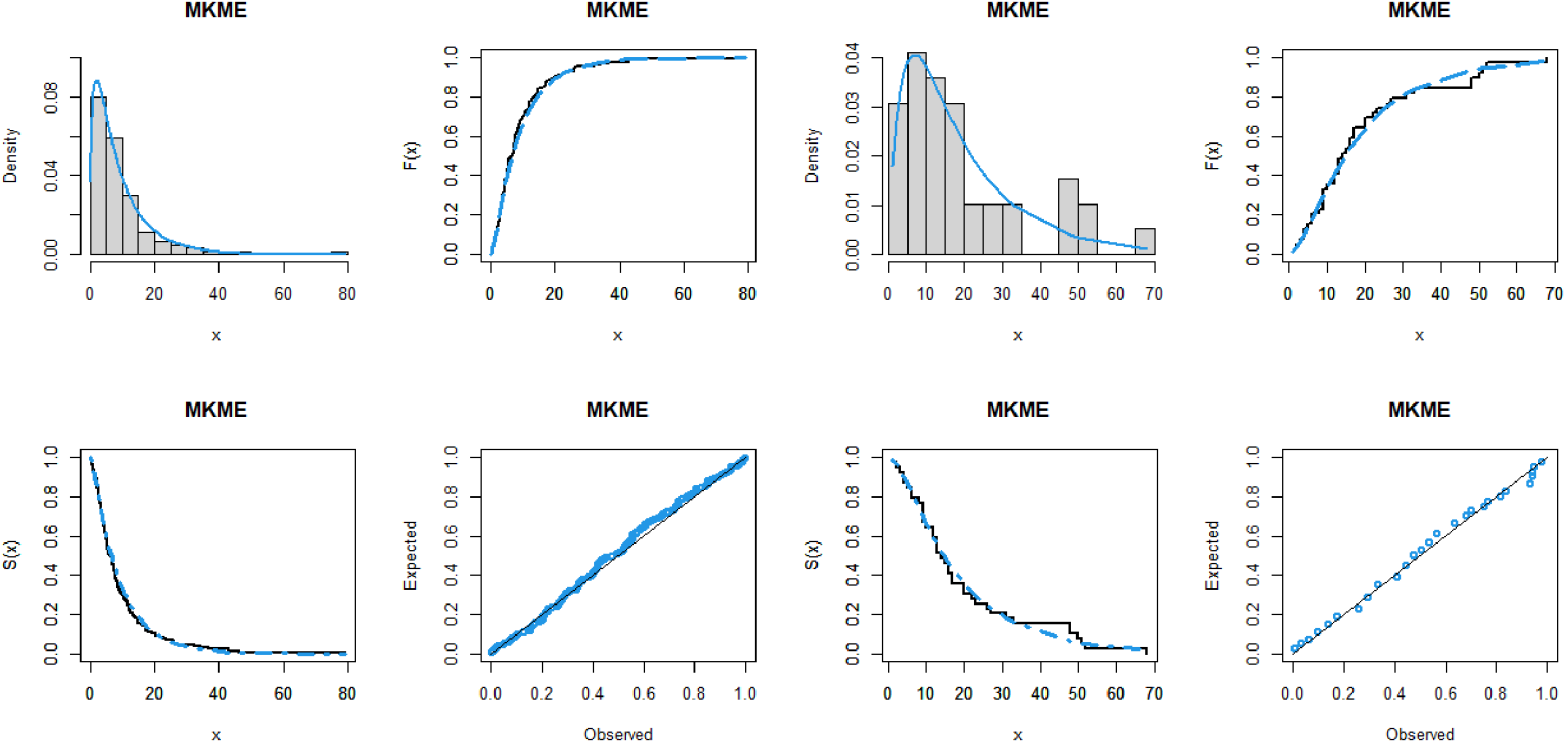
The fitted functions plots of the MKME model for cancer dataset (left panel) and vehicle fatalities dataset (right panel).

**Fig 10:**
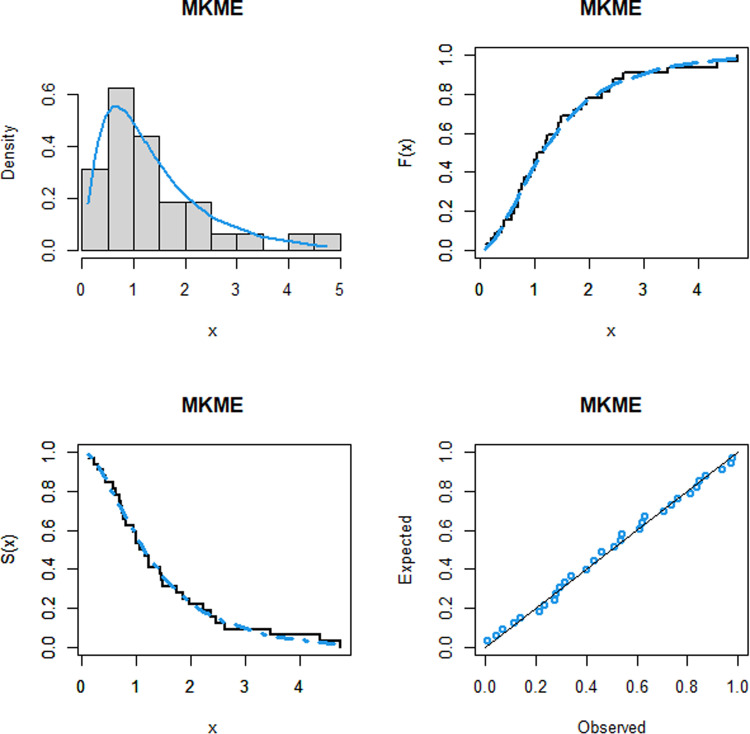
The fitted functions plot the MKME model for failures times dataset.

The values of the four measures, $W^{*}$, $A^{*}$, and KS as well as the *p*-value, of the fitted models are given in Tables Tables 7–11 for the five datasets, respectively. These tables also display the ML estimates and standard errors (SEs) of the parameters of the MKME distribution and other rival models. It is shown that, the MKME distribution has the lowest values of $W^{*}$, $A^{*}$, and KS statistics and largest *p*-value, showing its close fit to the five analyzed datasets. Furthermore, the fitting performance of the MKME model is explored visually through the plots of the PDF, CDF, SF, and probability-probability (PP) for all datasets. The plots are presented in Figs Fig 8, Fig 9, Fig 10. The plots indicate that the MKME provides the best fit to all datasets.

**Table 7:** The findings from waiting times dataset for the fitted distributions.

Distribution	Estimates (SEs)		$W^{*}$	$A^{*}$	KS	*p*-value
	*θ*	*λ*				
MKME	2.33584	0.13570	0.01721	0.12788	0.03669	0.99928
	(0.32997)	(0.01616)				
APE	21.17972	0.18310	0.06667	0.41794	0.05282	0.943031
	(14.17163)	(0.01975)				
GDUSE	1.85919	0.17644	0.03843	0.24227	0.04916	0.96908
	(0.31563)	(0.01853)				
GIE	7.85316	1.86621	0.27156	1.82525	0.10752	0.19797
	(0.93831)	(0.29240)				
GE	2.18364	0.15918	0.02079	0.14307	0.04023	0.99696
	(0.33431)	(0.01749)				
MOE	4.11667	0.19239	0.10730	0.65632	0.05966	0.86870
	(1.35417)	(0.02601)				
KME		0.07248	0.01757	0.12799	0.19201	0.00126
		(0.00822)				
E		0.10126	0.02709	0.17943	0.17304	0.00501
		(0.01012)				

**Table 8:** The findings from flood levels dataset for the fitted distributions.

Distribution	Estimates (SEs)		$W^{*}$	$A^{*}$	KS	*p*-value
	*θ*	*λ*				
MKME	47.15504	9.76075	0.04378	0.26702	0.11805	0.94321
	(31.6817)	(1.89813)				
APE	14860830.00	7.81324	0.05716	0.35991	0.15450	0.72622
	(19372.66125)	(0.63357)				
GDUSE	61.22504	12.00389	0.04991	0.31293	0.13107	0.88207
	(49.12459)	(2.20070)				
GIE	1.64479	37.70069	0.06908	0.43250	0.15834	0.69735
	(0.28876)	(23.19767)				
GE	57.57082	11.01567	0.04638	0.28609	0.12178	0.92814
	(42.06255)	(2.05639)				
MOE	480.38788	15.13971	0.09278	0.59425	0.14328	0.80614
	(600.29635)	(2.99146)				
KME		1.58063	0.06827	0.42657	0.45846	0.00045
		(0.40176)				
E		2.36322	0.07413	0.46196	0.46541	0.00036
		(0.52843)				

**Table 9:** The findings from cancer dataset for the fitted distributions.

Distribution	Estimates (SEs)		$W^{*}$	$A^{*}$	KS	*p*-value
	*θ*	*λ*				
MKME	1.37074	0.10300	0.06255	0.39010	0.05578	0.82064
	(0.15305)	(0.01244)				
APE	1.17443	0.11134	0.12830	0.76715	0.07932	0.39635
	(0.84384)	(0.02263)				
GDUSE	0.98799	0.13439	0.17399	1.02751	0.08480	0.31605
	(0.13463)	(0.01444)				
GIE	1.99445	0.746290	1.16155	6.86897	0.20669	0.0000
	(0.27046)	(0.08825)				
GE	1.21796	0.12117	0.11221	0.67412	0.07251	0.51132
	(0.14883)	(0.01357)				
MOE	1.05598	0.10987	0.12546	0.75108	0.08112	0.36865
	(0.32167)	(0.01986)				
KME		0.07970	0.07072	0.43844	0.10922	0.09433
		(0.00786)				
E		0.10677	0.11929	0.71598	0.08463	0.31844
		(0.00944)				

**Table 10:** The findings from vehicle fatalities dataset for the fitted distributions.

Distribution	Estimates (SEs)		$W^{*}$	$A^{*}$	KS	*p*-value
	*θ*	*λ*				
MKME	1.73224	0.05761	0.03430	0.25324	0.08843	0.92057
	(0.36692)	(0.01183)				
APE	4.91553	0.07315	0.07385	0.49020	0.10328	0.79977
	(4.95624)	(0.01637)				
GDUSE	1.30158	0.07456	0.06402	0.42882	0.10790	0.75427
	(0.33283)	(0.013521)				
GIE	9.45327	1.23876	0.19052	1.18516	0.18255	0.14859
	(1.98715)	(0.28642)				
GE	1.57264	0.06744	0.04646	0.32276	0.09291	0.88930
	(0.36160)	(0.01275)				
MOE	2.02223	0.07184	0.07787	0.50989	0.09920	0.83743
	(1.04004)	(0.01834)				
KME		0.03754	0.03788	0.27215	0.16357	0.24768
		(0.00679)				
E		0.05118	0.05051	0.34532	0.13834	0.44436
		(0.00819)				

**Table 11:** The findings from failures times dataset for the fitted distributions.

Distribution	Estimates (SEs)		$W^{*}$	$A^{*}$	KS	*p*-value
	*θ*	*λ*				
MKME	2.13564	0.88132	0.01548	0.11284	0.05791	0.99992
	(0.52357)	(0.18951)				
APE	13.99914	1.18216	0.04041	0.25898	0.08510	0.97460
	(16.16621)	(0.24167)				
GDUSE	1.66267	1.14361	0.03011	0.19518	0.08234	0.98175
	(0.49009)	(0.21785)				
GIE	0.99009	1.62935	0.12277	0.81223	0.14601	0.50258
	(0.21614)	(0.44070)				
GE	1.97235	1.03167	0.02094	0.14247	0.06810	0.99841
	(0.52412)	(0.205108)				
MOE	3.25667	1.19851	0.05296	0.33413	0.08370	0.97843
	(1.87575)	(0.30411)				
KME		0.49848	0.01817	0.12763	0.19958	0.156204
		(0.09978)				
E		0.69144	0.02463	0.16449	0.17874	0.25828
		(0.12223)				

### 7.2 Applications of the DMKME Model

In this subsection, we use two real count datasets to illustrate the flexibility of the DMKME distribution as compared to the existing discrete models including the transmuted record type geometric (TRTG) [33], exponentiated discrete Lindley (EDL) [34], natural discrete Lindley (NDL) [35], discrete Lindley (DL) [36], geometric (Gc), discrete Ramos-Louzada (DRL) [37], and discrete Poisson-Lindley (DPL) [38] distributions.

The first dataset represents the final marks of mathematics examination for 48 slow space students. This exam was done in the Indian Institute of Technology at Kanpur as discussed in [39]. The data are: 60, 29, 25, 50, 15, 13, 39, 27, 14, 15, 40, 18, 7, 7, 8, 19, 37, 12, 18, 5, 21, 15, 44, 86, 15, 14, 15, 70, 6, 50, 23, 58, 19, 23, 11, 6, 34, 18, 34, 12, 4, 28, 20, 23, 65, 19, 31, 21.

The second dataset represents the infant mortality rate per 1000 live births for some nations in 2021. The dataset is reported at https://data.worldbank.org/ indicator/SP.DYN.IMRT.IN. The data are: 12, 56, 10, 3, 22, 69, 7, 6, 11, 19, 44, 27, 13, 7, 12, 4, 3, 11, 27, 84, 25, 35, 6, 14, 6, 11.

Table 12 shows the ML estimates of the parameters of the discrete models and their corresponding SEs (in parenthesis) for the two count datasets. Furthermore, Table 12 provides the goodness-of-fit measures for all discrete models.

**Table 12:** The ML estimates and goodness-of-fit measures for the DMKME and other discrete models.

Count Data	Distribution	Estimates (SEs)		$W^{*}$	$A^{*}$	KS	*p*-value
Dataset I	DMKME	$\hat{\theta }$=2.8642 (0.6231)	$\hat{\lambda }$= 0.0575 (0.0096)	0.0469	0.2873	0.0675	0.9810
	TRTG	$\hat{q}$=0.9720 (0.0158)	$\hat{\theta }$= 0.9999 (0.5013)	0.0792	0.4534	0.0921	0.8102
	EDL	$\hat{\lambda }$= 0.9186 (0.0112)	$\hat{b}$= 1.3484 (0.3083)	0.0798	0.4595	0.0945	0.7851
	NDL	$\hat{\theta }$= 0.0695 (0.0069)		0.0798	0.4564	0.1006	0.7157
	DL	$\hat{\theta }$= 0.0732 (0.0075)		0.0773	0.4420	0.1021	0.6989
	Gc	$\hat{\theta }$= 0.0372 (0.0053)		0.0785	0.4495	0.2223	0.0174
	DRL	$\hat{\theta }$= 25.3057 (3.8133)		0.0788	0.4510	0.2224	0.0173
	DPL	$\hat{\theta }$= 0.0745 (0.0079)		0.0887	0.5086	0.1109	0.5966
Dataset II	DMKME	$\hat{\theta }$= 1.7582 (0.4675)	$\hat{\lambda }$=0.0543 (0.0139)	0.0932	0.5563	0.1395	0.6927
	TRTG	$\hat{q}$=0.9225 (0.0377)	$\hat{\theta }$= 0.7286 (0.8422)	0.1417	0.8468	0.1763	0.3940
	EDL	$\hat{\lambda }$=0.9239 (0.0161)	$\hat{b}$= 0.8007 (0.2270)	0.1337	0.7999	0.1747	0.4054
	NDL	$\hat{\theta }$= 0.0841 (0.0112)		0.1195	0.7140	0.2249	0.1431
	DL	$\hat{\theta }$= 0.0895 (0.0124)		0.1161	0.6934	0.2294	0.1295
	Gc	$\hat{\theta }$= 0.0456 (0.0087)		0.1193	0.7134	0.1703	0.4375
	DRL	$\hat{\theta }$= 20.3602 (4.2025)		0.1197	0.7159	0.1700	0.4399
	DPL	$\hat{\theta }$= 0.0916 (0.0133)		0.1339	0.8008	0.1937	0.2834

**Fig 11:**
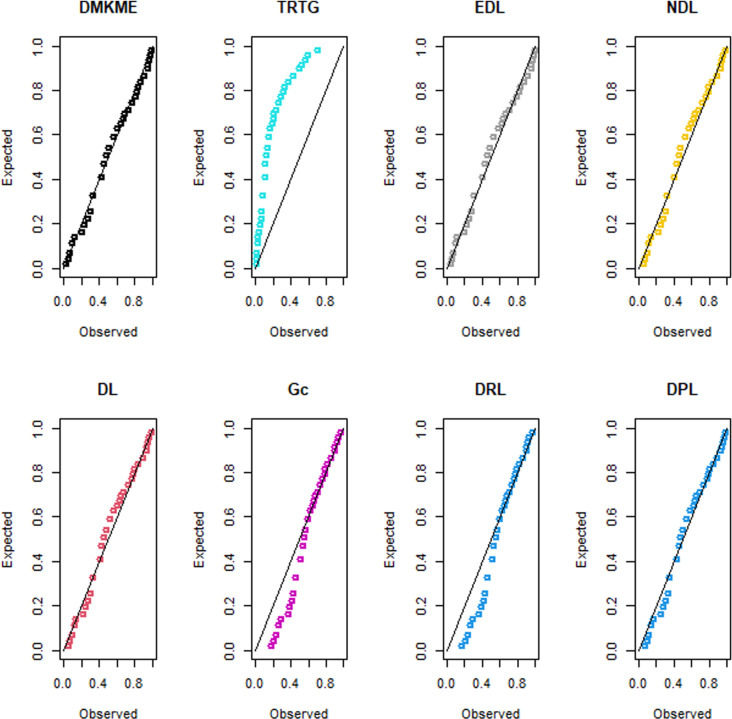
The PP plots of the fitted discrete models for dataset I.

**Fig 12:**
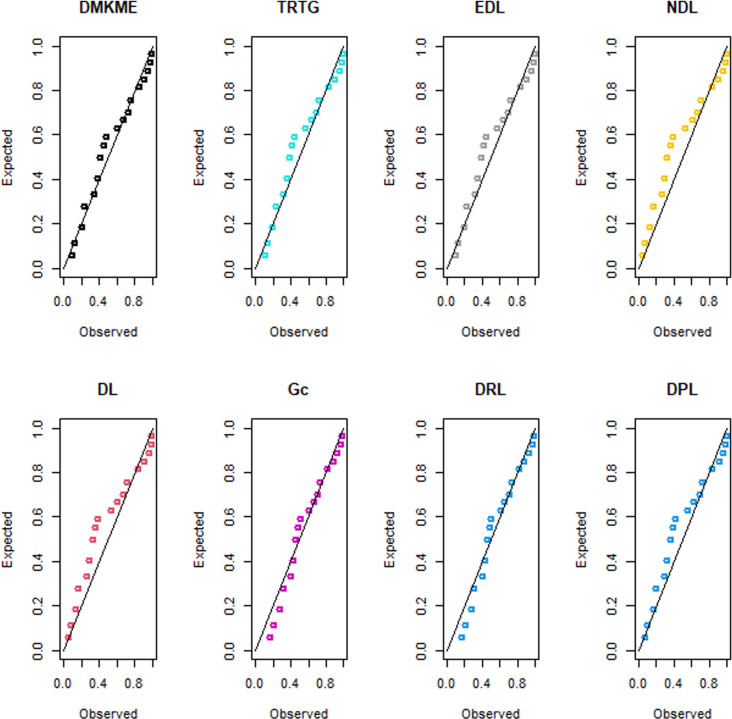
The PP plots of the fitted discrete models for dataset II.

The values in Table 12 indicate that the DMKME distribution outperforms all competing discrete models, with the lowest values for $W^{*}$, f $A^{*}$ and KS measures and the highest *p*-value, for the two count datasets. The PP plots for the two count datasets are displayed in Figs 11 and 12, respectively. These plots support the superior fit of the DMKME model, which provides a closer fit for both datasets as compared to other discrete distributions.

## 8 Some conclusions

We proposed two new classes of distributions called the modified Kavya-Manoharan-G (MKM-G) and discrete modified Kavya-Manoharan-G (DMKM-G) families. The MKM-G class extends and improves the flexibility of the Kavya-Manoharan (KM) family. Five special models of the MKM-G and DMKM-G families are provided. These special models have the advantage of being capable of modeling different shapes of aging and failure criteria. Basic mathematical properties of the MKM-G family are explored. We discuss eight approaches for estimating the parameters of the MKM-exponential (MKME) distribution, evaluating their performance through numerical simulations. We demonstrate that the MKME distribution provides a superior fit to five real-life datasets from the fields of reliability, medicine, engineering, and environmental science, outperforming several existing exponential models. Additionally, two count datasets are analyzed to illustrate the flexibility of the DMKM-exponential model. The newly discrete model provides better fits as compared to other important discrete distributions. Overall, the proposed models provide flexible and effective alternatives to existing distributions for modeling both lifetime and count data across diverse applied fields.

Future work on the MKM-G and DMKM-G families could include extending these models to bivariate and multivariate distributions for modeling dependent data in complex applications, as well as further generalizing them to incorporate more flexible hazard rate functions. Advanced estimation techniques, such as Bayesian or machine learning approaches, could be explored to improve accuracy in large datasets. The families could also be tested on big data from industries like finance, healthcare, and telecommunications. A comparative study with other flexible distribution families, the development of user-friendly software for implementation, and the extension to handle censored or truncated data are additional areas for future research.

## References

[1] Marshall A. A new method for adding a parameter to a family of distributions with application to the exponential and Weibull families. Biometrika. 1997;84(3):641–52. doi: 10.1093/biomet/84.3.641

[2] Cordeiro GM, de Castro M. A new family of generalized distributions. Journal of Statistical Computation and Simulation. 2011;81(7):883–98. doi: 10.1080/00949650903530745

[3] Bourguignon M, Silva RB, Cordeiro GM. The Weibull-G Family of Probability Distributions. Journal of Data Science. 2021;12(1):53–68. doi: 10.6339/jds.201401_12(1).0004

[4] Afify AZ, Cordeiro GM, Yousof HM, Alzaatreh A, Nofal ZM. The Kumaraswamy Transmuted-G Family of Distributions: Properties and Applications. Journal of Data Science. 2021;14(2):245–70. doi: 10.6339/jds.201604_14(2).0004

[5] Afify AZ, Alizadeh M. The odd Dagum family of distributions: Properties and applications. Journal of Applied Probability and Statistics. 2020;15(1):45–72.

[6] Zaidi SM, Sobhi MMA, El-Morshedy M, Afify AZ. A new generalized family of distributions: Properties and applications. MATH. 2021;6(1):456–76. doi: 10.3934/math.2021028

[7] Shama MS, El Ktaibi F, Al Abbasi JN, Chesneau C, Afify AZ. Complete Study of an Original Power-Exponential Transformation Approach for Generalizing Probability Distributions. Axioms. 2023;12(1):67. doi: 10.3390/axioms12010067

[8] Zhao Y, Ahmad Z, Alrumayh A, Yusuf M, Aldallal R, Elshenawy A, et al. A novel logarithmic approach to generate new probability distributions for data modeling in the engineering sector. Alexandria Engineering Journal. 2023;62:313–25. doi: 10.1016/j.aej.2022.07.021

[9] Abbasi JNA, Afify AZ, Alnssyan B, Shama MS. The Lambert-G Family: Properties, Inference, and Applications. CMES. 2024;140(1):513–36. doi: 10.32604/cmes.2024.046533

[10] Jamal F, Alqawba M, Altayab Y, Iqbal T, Afify AZ. A unified exponential-H family for modeling real-life data: Properties and inference. Heliyon. 2024;10(6):e27661. doi: 10.1016/j.heliyon.2024.e27661 38509929 PMC10951600

[11] Semary HE, Hussain Z, Hamdi WA, Aldahlan MA, Elbatal I, Nagarjuna VBV. Alpha–beta-power family of distributions with applications to exponential distribution. Alexandria Engineering Journal. 2024;100:15–31. doi: 10.1016/j.aej.2024.05.024

[12] Kavya P, Manoharan M. Some parsimonious models for lifetimes and applications. Journal of Statistical Computation and Simulation. 2021;91(18):3693–708. doi: 10.1080/00949655.2021.1946064

[13] Alotaibi N, Elbatal I, Shrahili M, Al-Moisheer AS, Elgarhy M, Almetwally EM. Statistical Inference for the Kavya–Manoharan Kumaraswamy Model under Ranked Set Sampling with Applications. Symmetry. 2023;15(3):587. doi: 10.3390/sym15030587

[14] Al-Nefaie AH. Applications to Bio-Medical data and statistical inference for a Kavya-Manoharan log-logistic model. Journal of Radiation Research and Applied Sciences. 2023;16(1):100523. doi: 10.1016/j.jrras.2023.100523

[15] Eldessouky EA, Hassan OHM, Elgarhy M, Hassan EAA, Elbatal I, Almetwally EM. A New Extension of the Kumaraswamy Exponential Model with Modeling of Food Chain Data. Axioms. 2023;12(4):379. doi: 10.3390/axioms12040379

[16] Hassan OHM, Elbatal I, Al-Nefaie AH, Elgarhy M. On the Kavya–Manoharan–Burr X Model: Estimations under Ranked Set Sampling and Applications. JRFM. 2022;16(1):19. doi: 10.3390/jrfm16010019

[17] Riad FH, Radwan A, Almetwally EM, Elgarhy M. A new heavy tailed distribution with actuarial measures. Journal of Radiation Research and Applied Sciences. 2023;16(2):100562. doi: 10.1016/j.jrras.2023.100562

[18] Swain JJ, Venkatraman S, Wilson JR. Least-squares estimation of distribution functions in johnson’s translation system. Journal of Statistical Computation and Simulation. 1988;29(4):271–97. doi: 10.1080/00949658808811068

[19] Cheng R, Amin N. Maximum product-of-spacings estimation with applications to the lognormal distribution. Mathematical Report. 1979;791.

[20] Cheng RCH, Amin NAK. Estimating Parameters in Continuous Univariate Distributions with a Shifted Origin. Journal of the Royal Statistical Society Series B: Statistical Methodology. 1983;45(3):394–403. doi: 10.1111/j.2517-6161.1983.tb01268.x

[21] Kao JHK. Computer Methods for Estimating Weibull Parameters in Reliability Studies. IRE Trans Reliab Qual Control. 1958;13:15–22. doi: 10.1109/ire-pgrqc.1958.5007164

[22] Cramér H. On the composition of elementary errors. Scandinavian Actuarial Journal. 1928;1928(1):13–74. doi: 10.1080/03461238.1928.10416862

[23] Von Mises RE. Wahrscheinlichkeit Statistik und Wahrheit. Switzerland: Springer; 1928.

[24] Ghitany ME, Atieh B, Nadarajah S. Lindley distribution and its application. Mathematics and Computers in Simulation. 2008;78(4):493–506. doi: 10.1016/j.matcom.2007.06.007

[25] Dumonceaux R, Antle CE. Discrimination Between the Log-Normal and the Weibull Distributions. Technometrics. 1973;15(4):923–6. doi: 10.1080/00401706.1973.10489124

[26] Lee ET, Wang JW. Statistical Methods for Survival Data Analysis. USA: John Wiley and Sons; 2003.

[27] Mann PS. Introductory Statistics. New York (USA): Wiley Publisher; 2016.

[28] Murthy DP, Xie M and Jiang R. Weibull Models. USA: John Wiley & Sons; 2004.

[29] Gupta RD, Kundu D. Theory & Methods: Generalized exponential distributions. Aus NZ J of Statistics. 1999;41(2):173–88. doi: 10.1111/1467-842x.00072

[30] Mahdavi A, Kundu D. A new method for generating distributions with an application to exponential distribution. Communications in Statistics - Theory and Methods. 2016;46(13):6543–57. doi: 10.1080/03610926.2015.1130839

[31] Maurya SK, Kaushik A, Singh SK, Singh U. A new class of distribution having decreasing, increasing, and bathtub-shaped failure rate. Communications in Statistics - Theory and Methods. 2016;46(20):10359–72. doi: 10.1080/03610926.2016.1235196

[32] Abouammoh AM, Alshingiti AM. Reliability estimation of generalized inverted exponential distribution. Journal of Statistical Computation and Simulation. 2009;79(11):1301–15. doi: 10.1080/00949650802261095

[33] Almazah MMA, Erbayram T, Akdoğan Y, AL Sobhi MM, Afify AZ. A New Extended Geometric Distribution: Properties, Regression Model, and Actuarial Applications. Mathematics. 2021;9(12):1336. doi: 10.3390/math9121336

[34] El-Morshedy M, Eliwa MS, Nagy H. A new two-parameter exponentiated discrete Lindley distribution: properties, estimation and applications. J Appl Stat. 2019;47(2):354–75. doi: 10.1080/02664763.2019.1638893 35706520 PMC9042015

[35] Al-Babtain AA, Ahmed AHN, Afify AZ. A New Discrete Analog of the Continuous Lindley Distribution, with Reliability Applications. Entropy (Basel). 2020;22(6):603. doi: 10.3390/e22060603 33286375 PMC7517138

[36] Bakouch HS, Jazi MA, Nadarajah S. A new discrete distribution. Statistics. 2012;48(1):200–40. doi: 10.1080/02331888.2012.716677

[37] Afify AZ, Elmorshedy M, Eliwa MS. A New Skewed Discrete Model: Properties, Inference, and Applications. Pak J Stat Oper Res. 2021;17:799–816. doi: 10.18187/pjsor.v17i4.3781

[38] Sankaran M. 275. Note: The Discrete Poisson-Lindley Distribution. Biometrics. 1970;26(1):145. doi: 10.2307/2529053

[39] Gupta RD, Kundu D. A new class of weighted exponential distributions. Statistics. 2009;43(6):621–34. doi: 10.1080/02331880802605346

